# Targeting Hypoxia: Hypoxia-Activated Prodrugs in Cancer Therapy

**DOI:** 10.3389/fonc.2021.700407

**Published:** 2021-07-29

**Authors:** Yue Li, Long Zhao, Xiao-Feng Li

**Affiliations:** ^1^Department of Nuclear Medicine, The Second Clinical Medical College, Jinan University (Shenzhen People’s Hospital), Shenzhen, China; ^2^The First Affiliated Hospital, Jinan University, Guangzhou, China; ^3^Department of Nuclear Medicine, The First Affiliated Hospital of Southern University of Science and Technology, Shenzhen, China

**Keywords:** hypoxia, hypoxia-activated prodrugs, tirapazamine, AQ4N, PR-104, EO9, TH-302, SN30000

## Abstract

Hypoxia is an important characteristic of most solid malignancies, and is closely related to tumor prognosis and therapeutic resistance. Hypoxia is one of the most important factors associated with resistance to conventional radiotherapy and chemotherapy. Therapies targeting tumor hypoxia have attracted considerable attention. Hypoxia-activated prodrugs (HAPs) are bioreductive drugs that are selectively activated under hypoxic conditions and that can accurately target the hypoxic regions of solid tumors. Both single-agent and combined use with other drugs have shown promising antitumor effects. In this review, we discuss the mechanism of action and the current preclinical and clinical progress of several of the most widely used HAPs, summarize their existing problems and shortcomings, and discuss future research prospects.

## Introduction

Hypoxia is a hallmark of a wide variety of solid tumors. In tumors, hypoxia arises due to a mismatch between oxygen delivery and consumption. Hypoxia is closely related to tumor progression, metastasis, therapeutic resistance, and poor prognosis (1). Hypoxia in tumor microenvironment leads to the transcriptional induction of a series of genes. The most important factor mediating this response is the hypoxia-inducible factor-1 (HIF-1), which extensively participates in glucose metabolism, angiogenesis, apoptosis, tumor metastasis and therapeutic resistance (2). Under hypoxic condition, HIF-1α regulates the switch from oxidative phosphorylation to anaerobic glycolysis, by activating the expression of glucose transporter 1 and 3 (GLUT-1 and GLUT-3) and related glycolytic enzymes (3). By regulating its downstream angiogenesis related genes, such as vascular endothelial growth factor (VEGF), basic fibroblast growth factor (bFGF), matrix metalloproteinases (MMPs), HIF-1α is widely involved in every step of angiogenesis, including endothelial progenitor cells recruitment and their differentiation to endothelial cells and smooth muscle cells, degradation of extracellular matrix, and the stability of peripheral cells (4). HIF-1α could induce apoptosis by regulating p53, Bcl-2, BNIP-3 and other genes (5). Through induction of MMPs, E-cadherin, CXCR4, CA9, HIF could promote tumor invasion and metastasis by regulating epithelial-to-mesenchymal transition (EMT) (6).

Tumor cells response to hypoxia depends in part on the duration of exposure. Hypoxic tumor cells may undergo necrosis, but some of the tumor cells may adjust to hypoxic stress and survive, which is also mediated by HIF-1α, resulting in a more aggressive phenotype and therapeutic resistance (5). Hypoxia and HIF could induce cell cycle arrest and hypoxic tumor cells generally have a relatively low proliferation rate (7, 8), while radiotherapy or chemotherapy mainly act on proliferating cells (9–11). Therefore, the hypoxic regions of tumors are usually insensitive to current radiotherapy and chemotherapy, and treatments targeting the hypoxic regions may provide additional clinical benefits. To this end, increasing efforts have been focused on the development of agents that selectively target and kill hypoxic tumor cells.

**Figure 1:**
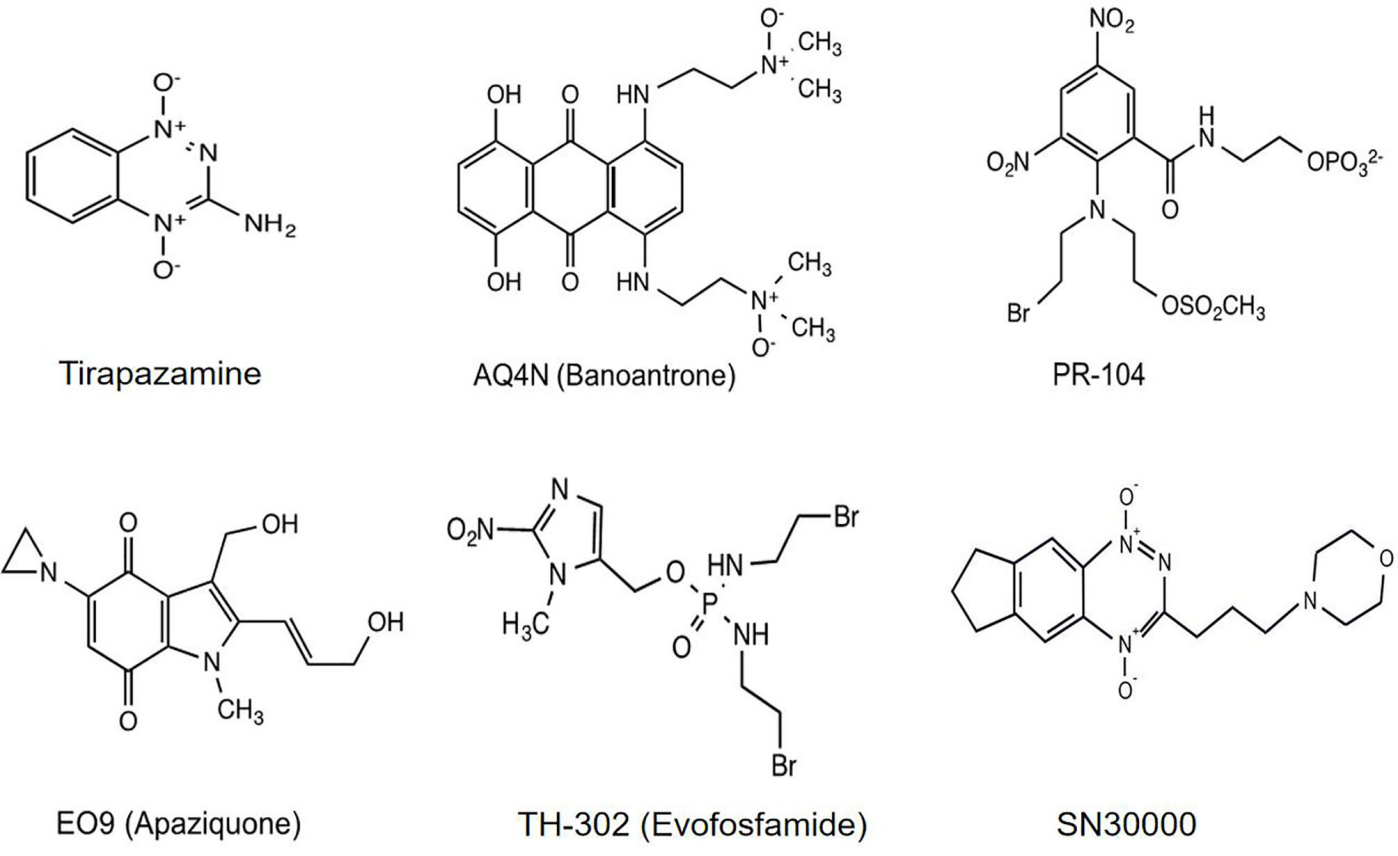
Chemical structures of representative HAPs.

Hypoxia-activated prodrugs (HAPs), also referred to as bioreductive drugs, are compounds that can be selectively reduced by specific reductases under hypoxic conditions to form cytotoxic agents that precisely target hypoxic tumor cells and have little toxicity to normal tissue. At present, several classes of HAPs have been developed, including quinones, nitroaromatics, aliphatic N-oxides and hetero-aromatic N-oxides. The most representative ones are tirapazamine, AQ4N (banoxantrone), PR-104, EO9 (apaziquone), TH-302 (evofosfamide), and SN30000 (**Figure 1**). This review puts a special emphasis on the past achievements as well as limitations of HAPs and attempts to analyze the potential reasons for unsuccessful clinical trials, with the aim of guiding future investigations into optimizing the use of this therapeutic approach.

## Tirapazamine

**Figure 2:**
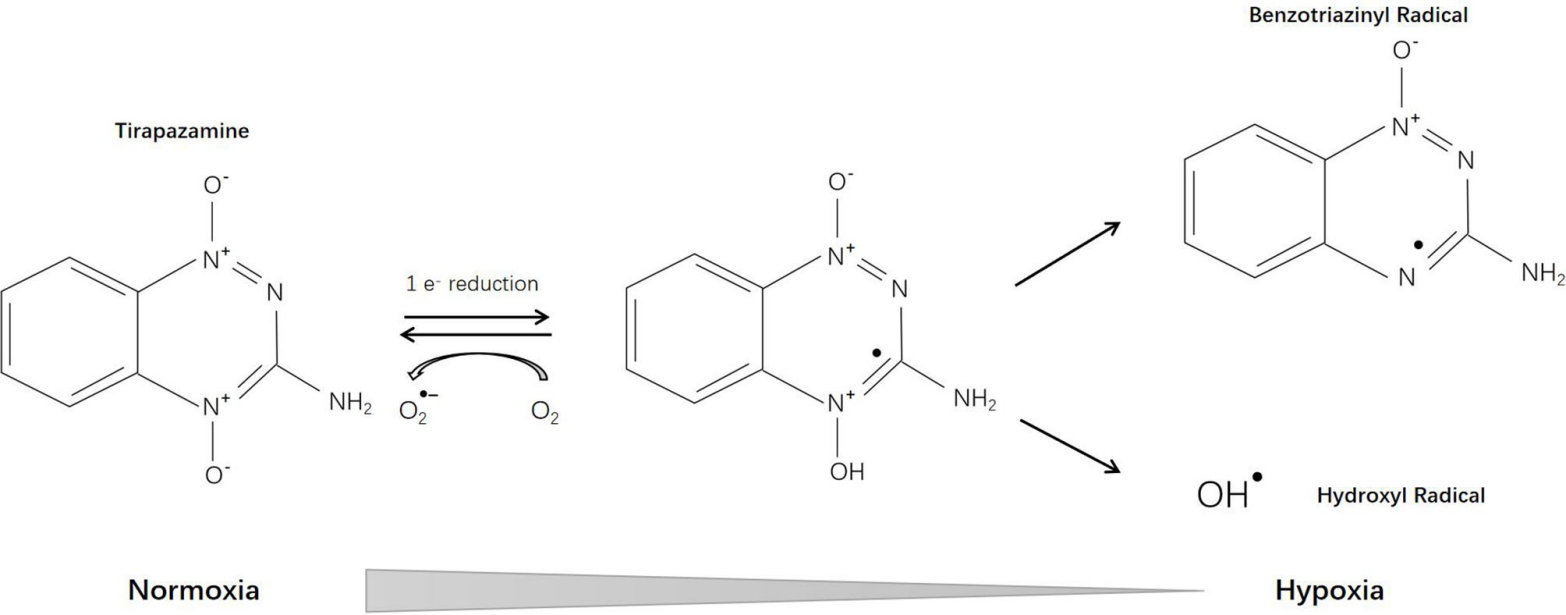
Reductive reaction of tirapazamine.

Tirapazamine (SR-4233, WIN 59075) [3-amino-1,2,4-benzotriazine-1,4 dioxide], the first hypoxia-activated prodrug, was reported in 1986 (12). Through one-electron reduction, the prodrug can generate an oxidative radical, which will diffuse into hypoxic regions and cause oxidative damage (13) (**Figure 2**). Cytochrome P-450 (CYP) is the main catalytic reductase involved in the reduction of tirapazamine (14). Although evidence showed that tirapazamine is a substrate for NAD(P)H: (quinone acceptor) oxidoreductase (DT-diaphorase) (15), the amount of DT-diaphorase expression in cells did not affect their sensitivity to tirapazamine (16).

Tirapazamine kills hypoxic cells by inducing chromosome aberrations and DNA double-strand breaks (17). Chromosome breaks caused by tirapazamine were more damaging and difficult to repair (18). Under hypoxic conditions, tirapazamine causes damage to both purine and pyrimidine residues in double-stranded DNA. DNA base damage was dominated by formation of formamidopyrimidine and 5-hydroxy-6-hydropyrimidine (19, 20). The DNA damaging activity of tirapazamine mainly results from radicals generated within the nucleus but not in the cytoplasm (21). Tirapazamine can induce acute changes in energy metabolism and intracellular pH in tumors (22). Skarsgard et al. (23) found that tirapazamine-induced DNA damage was pH-dependent (more effective at acidic pH) and could be repaired by certain gene products including uvrC and exonuclease III (24). The affinity of tirapazamine for hypoxic tissues was confirmed by many researchers but Durand and Olive demonstrated that this selectivity of tirapazamine was much lower *in vivo* (3 fold higher than aerobic) than that observed *in vitro* (50-500 fold) (25). Under aerobic conditions, tirapazamine can still induce cell cycle interruption and apoptosis, which may lead to its aerobic toxicity (26).

In preclinical studies, tirapazamine effectively inhibited tumor colony-forming *in vitro*, especially in hypoxic cells (27). Tirapazamine induced cell cycle arrest and apoptosis, and down-regulated HIF-1α, CA-IX and VEGF expression (28, 29). Brown (30) suggested that the activity of tirapazamine was p53-independent, but Yang’s study on neuroblastoma revealed that tirapazamine had clinical activity only in p53-functional neuroblastoma (31). Zeman and Brown published a series of reports focusing on the radiosensitization effects of tirapazamine. They reported that tirapazamine enhanced radiation-induced antineoplastic effects while sparing normal tissues (12, 32–38). As flavone acetic acid (FAA) reduces the blood supply of tumors, tirapazamine in combination with FAA could significantly enhance the antineoplastic efficacy of both drugs (39). Many studies have investigated the synergistic effect of tirapazamine and chemotherapy (such as cyclophosphamide, cisplatin, paclitaxel, etc.) or radioimmunotherapy (40–45). Tirapazamine, together with hyperthermia, electric pulses, etc. also exhibited encouraging antineoplastic efficacy (46–49). However, studies conducted by Adam et al. (50, 51) demonstrated that tirapazamine plus cisplatin and/or irradiation significantly increased toxicity and mortality.

In clinical trials, the reported adverse events associated with tirapazamine included muscle cramping, ototoxicity, granulocytopenia, nausea and vomiting, etc. (52, 53). Most phase 1 and 2 clinical trials have shown encouraging antineoplastic efficacy and tolerable toxicity (54–60). However, others, as well as two phase 3 clinical studies showed little benefit or significant toxicity (61–65).

## AQ4N

**Figure 3:**
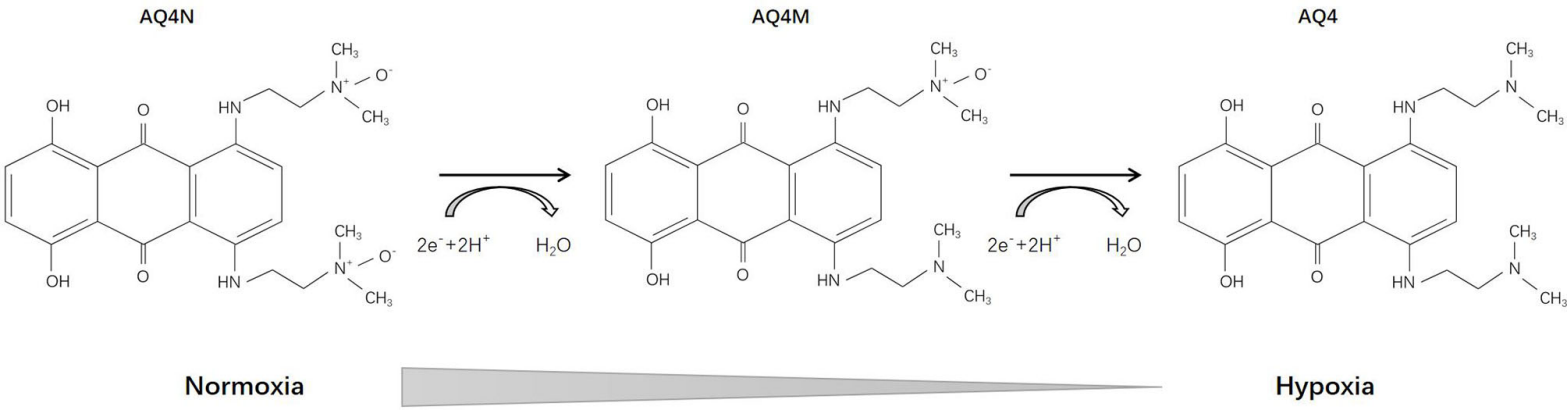
Reductive reaction of AQ4N.

AQ4N [1,4-bis{[2-(dimethylamino-N-oxide)ethyl]amino}-5,8-dihydroxyanthracene-9,10-dione], an aliphatic N-oxide, was first reported in 1993 (66). Its prodrug has no intrinsic DNA binding affinity and thus is non-toxic. Under hypoxic conditions, AQ4N can be activated into AQ4 (with an intermediate product AQ4M) through a two-electron reduction mediated by CYP, which is DNA-affinic and possesses 1000-fold cytotoxic potency compared with its prodrug (**Figure 3**). During the subsequent decade, Patterson and his team deeply investigated the pharmacology of AQ4N. They demonstrated that AQ4N combined with radiotherapy or chemotherapy (cisplatin, cyclophosphamide, thiotepa, mitoxantrone) showed enhanced antineoplastic effects (67–70). In 2003, they proposed a gene-directed enzyme prodrug therapy (GDEPT) strategy using CYPs in order to facilitate the bioreduction of AQ4N (71). Other researchers also investigated the activation of AQ4N by different types of CYPs and nitric oxide synthase (NOS) (72–75).

Many researchers have confirmed that AQ4N exerts antitumor effects in preclinical models of pancreatic cancer (76), bladder cancer and lung cancer (77), prostate cancer (78), gliosarcoma (79), etc., in both single-agent and combined chemotherapy, and in radiotherapy. Gieling et al. (80) demonstrated that AQ4N was more effective toward metastases in a fibrosarcoma-bearing mouse model (subcutaneous KHT tumors). Trédan et al. compared the penetration capacity of AQ4N and mitoxantrone through multi-layer cell cultures and tumor xenografts, and found that AQ4N could penetrate deeply into the hypoxic regions of the tumor and that combination therapy of AQ4N with mitoxantrone showed decreased tumor growth (81). There is also evidence showed that AQ4N had anti-angiogenic effects (82, 83).

The first phase 1 study of AQ4N was reported in 2007, in which 22 esophageal carcinoma patients received an AQ4N infusion followed by fractionated radiotherapy (84). Three of 22 patients had > 50% reductions in tumor volume and 9 had stable disease without dose-limiting toxicity. Albertella et al. enrolled 32 patients with different malignancies in a phase 1 study, and demonstrated that AQ4N was activated selectively in hypoxic regions of tumors and that it can penetrate the blood-brain barrier (85). No objective antitumor effect was observed in another phase 1 clinical study conducted by Papadopoulos et al. (86).

In recent years, a series of new therapeutic strategies have been under development, including combination therapy with AQ4N and photodynamic therapy (PDT), vascular-targeted photodynamic therapy (VTP) (87–92). Feng et al. (93) developed a treatment strategy that combined PDT with AQ4N. Using an AQ4N-64Cu-hCe6-liposome *in vivo* PET probe, they were able to monitor tumor hypoxia status after illumination with light-emitting diode light and demonstrated that utilization of PDT-induced hypoxia to trigger hypoxia-targeted therapy achieved significant antineoplastic effects. Zhang et al. (94) showed that AQ4N combined with starvation therapy (by using stealth liposomes to deliver glucose oxidase together with prodrugs) exhibited similar enhancement of antitumor effects. These methodologies provide new insights for future cancer diagnosis and therapy.

## PR-104

**Figure 4:**
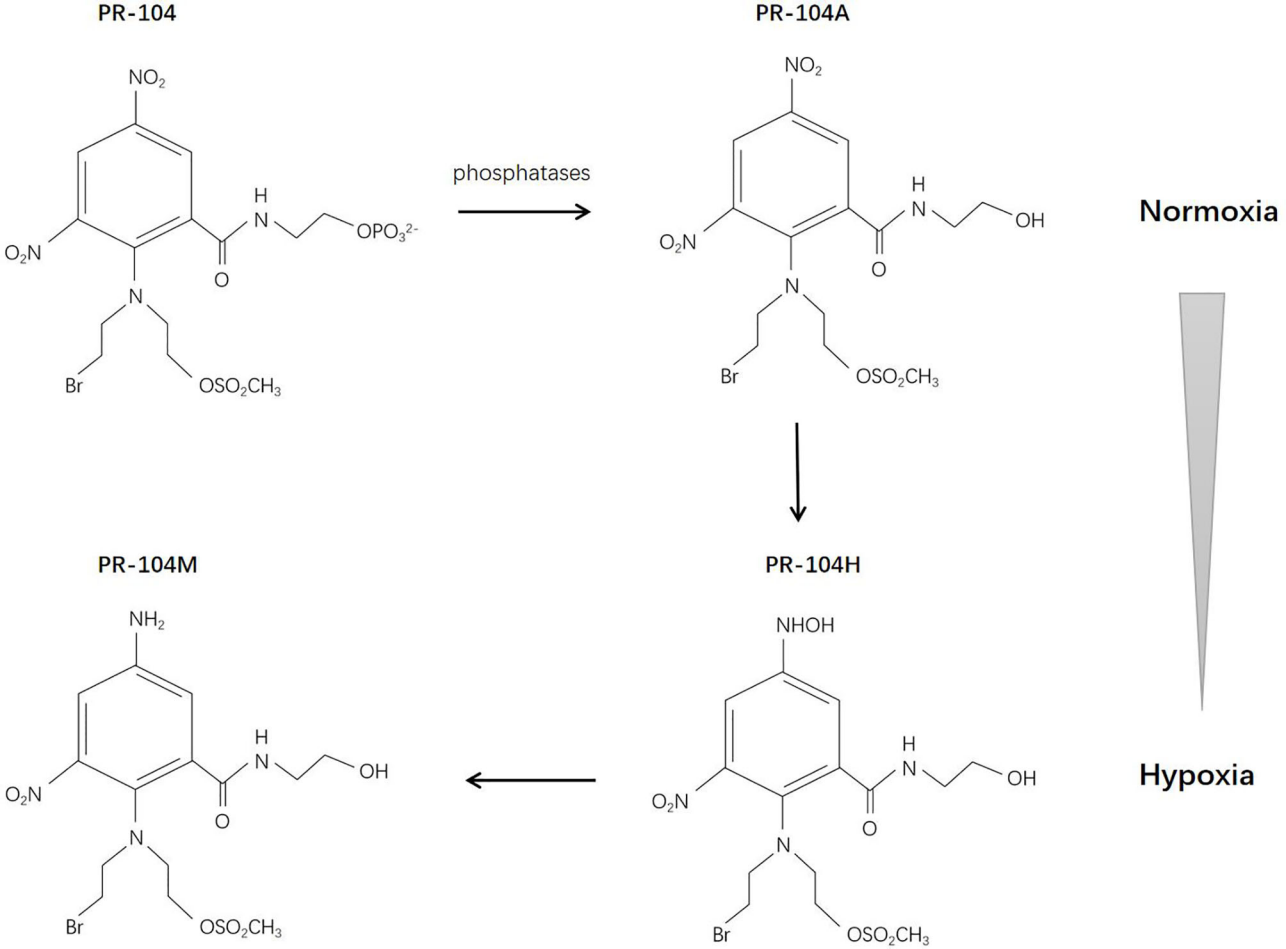
Reductive reaction of PR-104.

PR-104 is a 3,5-dinitrobenzamide-2-mustard. The water-soluble phosphate PR-104 can transform to a more lipophilic prodrug PR-104A (3,5-dinitrobenzamide-2-nitrogen mustard) systemically, and then, under hypoxic conditions, it can be further activated by reduction to PR-104H (5-hydroxylamine) and PR-104M (5-amine), allowing it to act as a DNA interstrand cross-linking agent in hypoxic cells and exert cytotoxic effects (95) (**Figure 4**). The reduction reaction is catalyzed anaerobically mainly by NADPH-cytochrome P450 reductase (96). There are studies demonstrating that PR-104 may also be reduced by aldo-keto reductase (AKR) 1C3 anaerobically, which might cause systemic toxicity (97, 98). The sensitivity of PR-104 depends on the oxygenation status, reductase activity, and DNA repair ability (99). Two studies have revealed the bystander effect of PR-104 (100, 101).

In *in vitro* studies, the antitumor efficacy of PR-104 has been investigated in cervical squamous cell carcinoma (SiHa cells), ovarian carcinoma (A2780 cells), non-small cell lung carcinoma (H1299 and A549 cells), colorectal carcinoma (RKO and HCT116 cells), hepatocellular carcinoma, etc., PR-104 as a single agent or in combination with radiotherapy or chemotherapy has shown different degrees of antineoplastic effects (95, 102–104).

In clinical trials, however, no or only partial responses were observed, but with obvious toxicities, mainly thrombocytopenia and neutropenia (105–108). However, PR-104 showed advantages in the treatment of leukemia. Evidence showed that in acute lymphoblastic leukemia, T-cell acute lymphoblastic leukemia, and acute myeloid leukemia, PR-104 decreased tumor burden and prolonged survival in pre-clinical studies (109), and also was associated with disease response in a phase I/II clinical trial (110). The expression of AKR1C3 can be used as a biomarker to predict response to PR-104 and patients screening (111).

## EO9 (Apaziquone)

**Figure 5:**
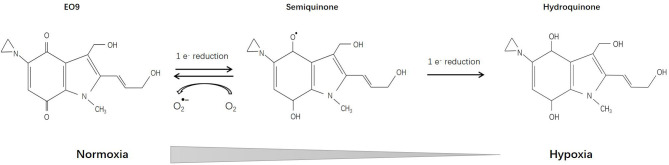
Reductive reaction of EO9.

EO9 (Apaziquone) [3-hydroxy-5-aziridinyl-1-methyl-2(1H-indole-4,7-dione)prop-beta-en-alpha-ol], which is structurally related to mitomycin C, was first reported 1989 and has been deeply investigated since then. Pharmacological studies have shown that DT-diaphorase plays a vital role in the reduction of EO9 prodrug (112), implying that detection of DT-diaphorase activity might predict the sensitivity of certain tumors to EO9 (113–115) (**Figure 5**).

*In vitro*, EO9 was proved effective toward colon adenocarcinoma cells, melanoma cells, central nervous system tumors, renal cancer cells, oral squamous cell carcinoma, and lung cancer cells (including NSCLC and certain cell lines of small cell lung cancer). *In vivo*, gastric and colorectal adenocarcinoma, ovarian carcinoma, and breast carcinoma were sensitive while leukemia was found to be resistant to EO9 (116–118). Certain inducers such as 1,2-dithiole-3-thiones (D3T) could enhance DT-diaphorase activity, thereby increasing the sensitivity of EO9 (119, 120). However, some researchers pointed out that *in vitro* studies on DT-diaphorase activity are different from *in vivo* studies, and may result in different sensitivity measurements (121). Further pharmacological studies have shown that in the presence of oxygen, DT-diaphorase reduces EO9 through 2-electron reduction, and the product is hydroquinone; while under hypoxic conditions, EO9 undergoes 1-electron reduction, and the product is semiquinone, which is more toxic than hydroquinone (122, 123). Therefore, EO9 may be more effective for hypoxic solid tumors (124, 125). Studies have also shown that the anti-tumor effect of EO9 is pH-dependent, and may exert a tumor suppressor effect in tumor areas with low pH (pH5.5-7.0) (126).

For clinical trials, nephrotoxicity and proteinuria were observed in both phase 1 and phase 2 clinical studies, but only partial response or stable disease was achieved (127–131). The reason for these unsatisfactory results may be attributed to the instability of both semiquinone and hydroquinone, with a short half-life and poor permeability, which will be quickly removed *in vivo* (131–134). However, this special pharmacokinetic profile is ideal for local treatment (135, 136). Intravesical instillation of EO9 was well tolerated and effective for superficial bladder cancer, manifested by a higher complete remission rate and a lower recurrence rate (137–139). A recent study pointed out that EO9 may be inactivated by hematuria, which suggests that the timing of medication should be selected with this in mind in the design of future phase 3 clinical trials (140).

## TH-302 (Evofosfamide)

**Figure 6:**
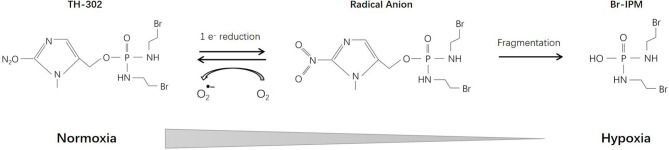
Reductive reaction of TH-302.

TH-302 (Evofosfamide), a second-generation HAP, consists of a 2-nitroimidazole moiety linked to bromo-iso-phosphoramide mustard (Br-IPM). Br-IPM is a DNA cross-linking agent. Under hypoxic conditions through a 2-nitroimidazole reduction reaction, TH-302 prodrug releases Br-IPM and perform cytotoxic effect (141) (**Figure 6**). Cytochrome P450 oxidoreductase (POR) also plays an important role in the reduction reaction and is the main determinant of cell sensitivity to TH-302 (142). Thus, the efficacy of TH-302 is highly dependent on the tumor type (143).

Many researchers have reported the antitumor efficacy of TH-302 as a single agent in malignancies including multiple myeloma, osteosarcoma, chondrosarcoma, neuroblastoma, rhabdomyosarcoma, breast cancer, non-small cell lung cancer, head and neck tumors, acute myeloid leukemia, etc. (144–152). The effect of TH-302 on spherical cells was significantly enhanced (153) and its activity was related to tumor hypoxic fractions (154), indicating that TH-302 had high hypoxic selectivity. The reported antineoplastic mechanisms include DNA fragmentation, cell cycle arrest, down-regulation of hypoxia-inducible factor-1α expression, etc.

In addition to monotherapy, TH-302 also showed synergistic effects with many traditional chemotherapy drugs, including doxorubicin, topotecan, paclitaxel, cisplatin, docetaxel, pemetrexed, irinotecan, gemcitabine, and temozolomide (155–157). TH-302 was able to inhibit the reoxygenation and proliferation of hypoxic tumor cells that survived chemotherapy (158). Studies also revealed that the application of hypoxia inducers, such as Chk1 inhibitor, mTOR inhibitor, hydralazine, and pyruvate, enhanced the efficacy of TH-302 (159–161). TH-302 also has a radiosensitization effect. It exerts a synergistic effect when combined with radiotherapy (162–164). TH-302 has been shown to be beneficial in combination with conventional transarterial chemoembolization (cTACE) (165); anti-angiogenic therapy, such as VEGF-A inhibitor, sunitinib, and pazopanib (166–168); molecular targeted therapy, such as sorafenib and erlotinib (169, 170); and immunotherapy, such as CTLA-4 and PD-1 blockade (171, 172), where it also exerted a significant tumor inhibition effect. Recent evidence suggests that TH-302 can not only kill hypoxic pancreatic cancer cells, but also has the ability to improve the oxygenation status of residual tumor cells, so it may be useful to enhance the effect of radiotherapy and chemotherapy (173).

Since 2007, TH-302 has been in clinical trials. The main toxicities reported were skin and/or mucosal toxicity, thrombocytopenia, neutropenia, and myelosuppression (174–177). Several phase 1/2 clinical trials have reported encouraging results. For several types of tumors, including soft tissue sarcoma, pancreatic cancer, glioblastoma, and papillomavirus-negative head and neck squamous cell carcinoma, etc, TH-302 alone or in combination with other therapies showed varying degrees of antineoplastic activity (171, 175–178). It showed limited efficacy in the treatment of leukemia and failed in two phase 3 clinical trials (179–181). Researchers analyzed the possible reasons, including the lack of patient screening based on tumor hypoxia status (182, 183), antagonism between drugs (184), and drug formulation changes (185). Further research is still in progress.

## SN30000

**Figure 7:**
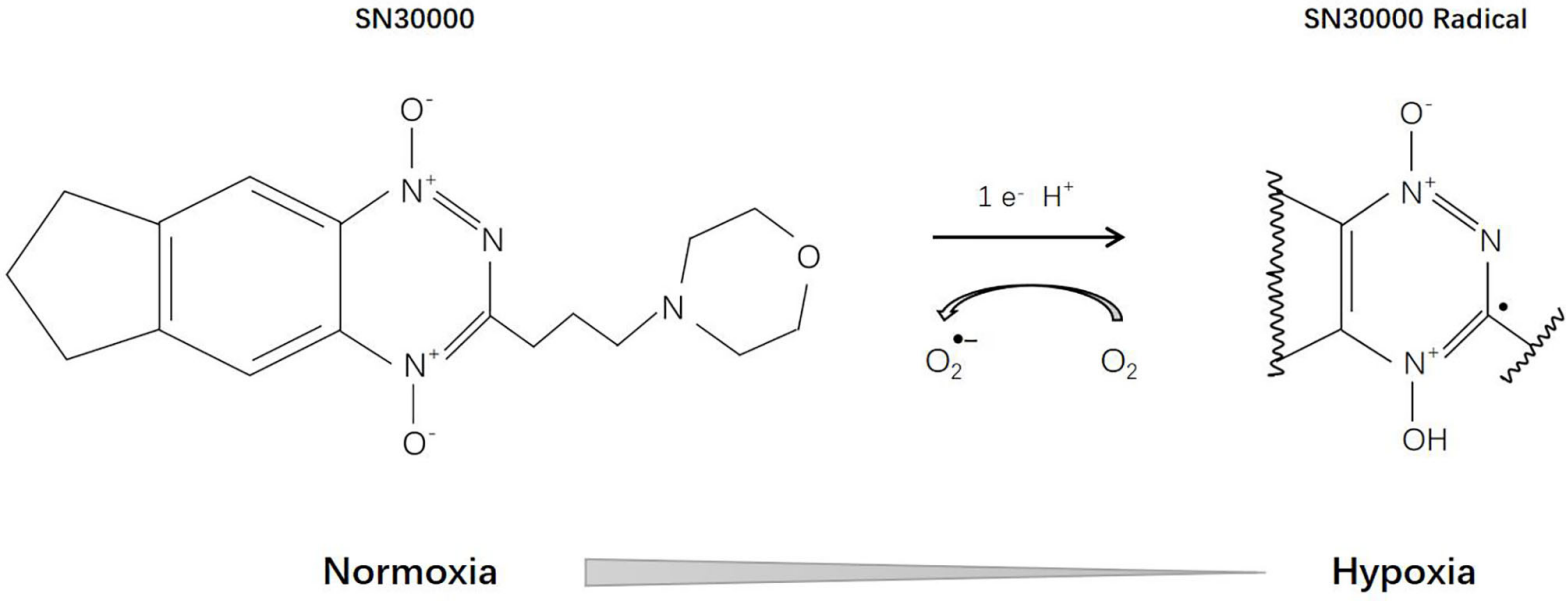
Reductive reaction of SN30000.

SN30000 [3-(3-Morpholinopropyl)-7,8-dihydro-6H-indeno[5,6-e][1,2,4]triazine 1,4-dioxide], previously known as CEN-209, is a second-generation benzotriazine-N-oxide hypoxia-activated prodrug and a modified analogue of tirapazamine (**Figure 7**). Currently, it is still in the stage of preclinical research. Several studies have confirmed that SN30000 possesses similar pharmacological mechanisms (186) to tirapamine, but is superior in terms of antineoplastic effects and hypoxia selectivity (187).

Mao et al. (188) proved that, compared with monolayer tumor cells, SN30000 has higher activity on tumor spheroids, and when combined with radiation, it can cause significant tumor spheroid growth delay. Moreover, when used together with or before gemcitabine, SN30000 can effectively inhibit the proliferation of reoxygenated tumor cells (189). EF5 binding may be a promising biomarker for hypoxia stratification and SN30000 treatment response assessment (190, 191).

## Conclusions and Suggestions for Future Investigations

Since the 1980s, HAPs have been developed and validated step by step, from preclinical to clinical. Despite their antineoplastic effects, their drawbacks and limitations have also been revealed by many studies. Here, we summarize the past experience and the latest research progress, and propose the following directions for future research (**Table 1**):

**Table 1:** Summary points.

Summary points
Current Research Status: •At present, the research of HAPS is mostly limited to the curative effect of macroscopic solid tumors. However, the results are not satisfactory. •Evidence showed that hypoxic tumor cells could only survive for 2-3 days *in vivo*, suggesting that *in vivo* hypoxic cells are destined to enter necrosis *in vivo* and that hypoxia-targeting therapy of macroscopic tumors should be revisited.
Suggestions For Future Investigations: •Our experimental evidence showed that micro-metastases (< 1 mm in diameter) and tumor cells in ascites and pleural effusion were severely hypoxic and in low proliferation state. •They were insensitivity to traditional radiotherapy and chemotherapy. •Micro-metastases (< 1 mm in diameter) and tumor cells in ascites and pleural effusion are more suitable therapeutic targets for HAPs.

First, screening methods need to be developed based on tumor hypoxia to select the best candidates for this type of therapy. A growing number of studies have shown that PET/CT imaging can be an effective method to monitor HAPs uptake and therapeutic response (148, 190, 192). Second, biomarkers to predict drug sensitivity are needed. Since HAP is a bioreductive drug, it requires specific enzymes to complete the reduction reaction. Therefore, the detection of specific enzymes can play a role in predicting drug sensitivity (112, 142). In addition, experiments conducted by our group and others showed that hypoxic tumor cells could only survive for 2-3 days *in vivo* (193, 194), suggesting that *in vivo* hypoxic cells are destined to enter necrosis *in vivo* and that hypoxia-targeting therapy of macroscopic tumors should be revisited.

Hypoxia is not only a characteristic of macroscopic tumors. In 2007, Li et al. reported that peritoneal disseminated micro-metastases (< 1 mm in diameter) were severely hypoxic and in low proliferation state (7, 8, 195–197). This hypoxic state of early micrometastases likely confers insensitivity to traditional radiotherapy and chemotherapy, making them suitable therapeutic targets for HAPs. HAPs may have the potential to prevent them from developing into macroscopic tumors, thereby reducing the metastatic rate of tumors. Our group is working to further confirm the efficacy of HAPs on such tumors and its effect on early tumor metastasis.

## Data Availability Statement

The original contributions presented in the study are included in the article/supplementary files. Further inquiries can be directed to the corresponding author.

## Author Contributions

YL performed the literature search and wrote the manuscript. LZ performed the literature search and figure editing. X-FL contributed to write and revise the manuscript. All authors contributed to the article and approved the submitted version.

## Conflict of Interest

The authors declare that the research was conducted in the absence of any commercial or financial relationships that could be construed as a potential conflict of interest.

## Publisher’s Note

All claims expressed in this article are solely those of the authors and do not necessarily represent those of their affiliated organizations, or those of the publisher, the editors and the reviewers. Any product that may be evaluated in this article, or claim that may be made by its manufacturer, is not guaranteed or endorsed by the publisher.

## References

[1] Evans SM Koch CJ. Prognostic Significance of Tumor Oxygenation in Humans. Cancer Lett (2003) 195(1):1–16. 10.1016/s0304-3835(03)00012-0 12767506

[2] Challapalli A Carroll L Aboagye EO. Molecular Mechanisms of Hypoxia in Cancer. Clin Transl Imaging (2017) 5(3):225–53. 10.1007/s40336-017-0231-1 PMC5437135 28596947

[3] Bristow RG Hill RP. Hypoxia and Metabolism. Hypoxia, DNA Repair and Genetic Instability. Nat Rev Cancer (2008) 8(3):180–92. 10.1038/nrc2344 18273037

[4] Carmeliet P Jain RK. Molecular Mechanisms and Clinical Applications of Angiogenesis. Nature (2011) 19 473(7347):298–307. 10.1038/nature10144 PMC4049445 21593862

[5] Lum JJ Bui T Gruber M Gordan JD DeBerardinis RJ Covello KL. The Transcription Factor HIF-1alpha Plays a Critical Role in the Growth Factor-Dependent Regulation of Both Aerobic and Anaerobic Glycolysis. Genes Dev (2007) 21(9):1037–49. 10.1101/gad.1529107 PMC1855230 17437992

[6] Joseph JP Harishankar MK Pillai AA Devi A. Hypoxia Induced EMT: A Review on the Mechanism of Tumor Progression and Metastasis in OSCC. Oral Oncol (2018) 80:23–32. 10.1016/j.oraloncology.2018.03.004 29706185

[7] Li XF Carlin S Urano M Russell J Ling CC O’Donoghue JA. Visualization of Hypoxia in Microscopic Tumors by Immunofluorescent Microscopy. Cancer Res (2007) 67(16):7646–53. 10.1158/0008-5472.CAN-06-4353 17699769

[8] Li XF O’Donoghue JA. Hypoxia in Microscopic Tumors. Cancer Lett (2008) 264(2):172–80. 10.1016/j.canlet.2008.02.037 PMC2460565 18384940

[9] Horsman MR Overgaard J. The Impact of Hypoxia and Its Modification of the Outcome of Radiotherapy. J Radiat Res (2016) 57(Suppl 1):i90–8. 10.1093/jrr/rrw007 PMC4990104 26983987

[10] Manoochehri Khoshinani H Afshar S Najafi R. Hypoxia: A Double-Edged Sword in Cancer Therapy. Cancer Invest (2016) 34(10):536–45. 10.1080/07357907.2016.1245317 27824512

[11] Kim JY Lee JY. Targeting Tumor Adaption to Chronic Hypoxia: Implications for Drug Resistance, and How it Can be Overcome. Int J Mol Sci (2017) 18(9):1854. 10.3390/ijms18091854 PMC5618503 28841148

[12] Zeman EM Brown JM Lemmon MJ Hirst VK Lee WW. Sr-4233: A New Bioreductive Agent With High Selective Toxicity for Hypoxic Mammalian Cells. Int J Radiat Oncol Biol Phys (1986) 12(7):1239–42. 10.1016/0360-3016(86)90267-1 3744945

[13] Laderoute K Wardman P Rauth AM. Molecular Mechanisms for the Hypoxia-Dependent Activation of 3-Amino-1,2,4-Benzotriazine-1,4-Dioxide (Sr 4233). Biochem Pharmacol (1988) 37(8):1487–95. 10.1016/0006-2952(88)90010-x 3128984

[14] Saunders MP Patterson AV Chinje EC Harris AL Stratford IJ. NADPH: Cytochrome C (P450) Reductase Activates Tirapazamine (SR4233) to Restore Hypoxic and Oxic Cytotoxicity in an Aerobic Resistant Derivative of the A549 Lung Cancer Cell Line. Br J Cancer (2000) 82(3):651–6. 10.1054/bjoc.1999.0977 PMC2363339 10682679

[15] Riley RJ Workman P. Enzymology of the Reduction of the Potent Benzotriazine-di-N-oxide Hypoxic Cell Cytotoxin SR 4233 (WIN 59075) by NAD(P)H: (Quinone Acceptor) Oxidoreductase (EC 1.6.99.2) Purified From Walker 256 Rat Tumour Cells. Biochem Pharmacol (1992) 43(2):167–74. 10.1016/0006-2952(92)90274-m 1739405

[16] Patterson AV Robertson N Houlbrook S Stephens MA Adams GE Harris AL. The Role of DT-diaphorase in Determining the Sensitivity of Human Tumor Cells to Tirapazamine (Sr 4233). Int J Radiat Oncol Biol Phys (1994) 29(2):369–72. 10.1016/0360-3016(94)90291-7 8195035

[17] Elwell JH Siim BG Evans JW Brown JM. Adaptation of Human Tumor Cells to Tirapazamine Under Aerobic Conditions: Implications of Increased Antioxidant Enzyme Activity to Mechanism of Aerobic Cytotoxicity. Biochem Pharmacol (1997) 54(2):249–57. 10.1016/s0006-2952(97)00171-8 9271329

[18] Wang J Biedermann KA Brown JM. Repair of DNA and Chromosome Breaks in Cells Exposed to SR 4233 Under Hypoxia or to Ionizing Radiation. Cancer Res (1992) 52(16):4473–7. 10.1002/1097-0142(19920815)70:4<903::AID-CNCR2820700432>3.0.CO;2 1643639

[19] Kotandeniya D Ganley B Gates KS. Oxidative DNA Base Damage by the Antitumor Agent 3-amino-1,2,4-benzotriazine 1,4-Dioxide (Tirapazamine). Bioorg Med Chem Lett (2002) 12(17):2325–9. 10.1016/s0960-894x(02)00468-7 12161126

[20] Birincioglu M Jaruga P Chowdhury G Rodriguez H Dizdaroglu M Gates KS. Dna Base Damage by the Antitumor Agent 3-amino-1,2,4-benzotriazine 1,4-Dioxide (Tirapazamine). J Am Chem Soc (2003) 125(38):11607–15. 10.1021/ja0352146 13129365

[21] Evans JW Yudoh K Delahoussaye YM Brown JM. Tirapazamine Is Metabolized to Its DNA-Damaging Radical by Intranuclear Enzymes. Cancer Res (1998) 58(10):2098–101. 9605751

[22] Aboagye EO Dillehay LE Bhujwalla ZM Lee DJ. Hypoxic Cell Cytotoxin Tirapazamine Induces Acute Changes in Tumor Energy Metabolism and Ph: A 31p Magnetic Resonance Spectroscopy Study. Radiat Oncol Investig (1998) 6(6):249–54. 10.1002/(SICI)1520-6823(1998)6:6<249::AID-ROI1>3.0.CO;2-C 9885940

[23] Skarsgard LD Vinczan A Skwarchuk MW Chaplin DJ. The Effect of Low Ph and Hypoxia on the Cytotoxic Effects of SR4233 and Mitomycin C *In Vitro* . Int J Radiat Oncol Biol Phys (1994) 29(2):363–7. 10.1016/0360-3016(94)90290-9 8195034

[24] Koch CJ. Unusual Oxygen Concentration Dependence of Toxicity of SR-4233, a Hypoxic Cell Toxin. Cancer Res (1993) 53(17):3992–7. 10.1007/BF01518522 8358728

[25] Durand RE Olive PL. Physiologic and Cytotoxic Effects of Tirapazamine in Tumor-Bearing Mice. Radiat Oncol Investig (1997) 5(5):213–9. 10.1002/(SICI)1520-6823(1997)5:5<213::AID-ROI1>3.0.CO;2-0 9372543

[26] Lin PS Ho KC Yang SJ. Tirapazamine (SR 4233) Interrupts Cell Cycle Progression and Induces Apoptosis. Cancer Lett (1996) 105(2):249–55. 10.1016/0304-3835(96)04292-9 8697451

[27] Hanauske AR Ross M Degen D Hilsenbeck SG Von Hoff DD. *In Vitro* Activity of the Benzotriazine Dioxide SR 4233 Against Human Tumour Colony-Forming Units. Eur J Cancer (1993) 29A(3):423–5. 10.1016/0959-8049(93)90400-a 8398345

[28] Hong B Lui VW Hui EP Ng MH Cheng SH Sung FL. Hypoxia-Targeting by Tirapazamine (Tpz) Induces Preferential Growth Inhibition of Nasopharyngeal Carcinoma Cells With Chk1/2 Activation. Invest New Drugs (2011) 29(3):401–10. 10.1007/s10637-009-9356-z 20013349

[29] Govaert KM Nijkamp MW Emmink BL Steller EJ Minchinton AI Kranenburg O. Effects of Tirapazamine on Experimental Colorectal Liver Metastases After Radiofrequency Ablation. Br J Surg (2012) 99(4):567–75. 10.1002/bjs.8668 22331808

[30] Brown JM. Exploiting Tumour Hypoxia and Overcoming Mutant P53 With Tirapazamine. Br J Cancer (1998) 77(Suppl 4):12–4. 10.1038/bjc.1998.430 PMC2149884 9647614

[31] Yang B Reynolds CP. Tirapazamine Cytotoxicity for Neuroblastoma is p53 Dependent. Clin Cancer Res (2005) 11(7):2774–80. 10.1158/1078-0432.CCR-04-2382 15814660

[32] Brown JM Lemmon MJ. Potentiation by the Hypoxic Cytotoxin SR 4233 of Cell Killing Produced by Fractionated Irradiation of Mouse Tumors. Cancer Res (1990) 50(24):7745–9. 10.1002/1097-0142(19901215)66:12<2673::AID-CNCR2820661235>3.0.CO 2253217

[33] Minchinton AI Brown JM. Enhancement of the Cytotoxicity of SR 4233 to Normal and Malignant Tissues by Hypoxic Breathing. Br J Cancer (1992) 66(6):1053–8. 10.1038/bjc.1992.409 PMC1978042 1457345

[34] el-Said A Menke D Dorie MJ Brown JM. Comparison of the Effectiveness of Tirapazamine and Carbogen With Nicotinamide in Enhancing the Response of a Human Tumor Xenograft to Fractionated Irradiation. Radiat Oncol Investig (1999) 7(3):163–9. 10.1002/(SICI)1520-6823(1999)7:3<163::AID-ROI5>3.0.CO;2-M 10406058

[35] Shibata T Shibamoto Y Sasai K Oya N Murata R Takagi T. Comparison of *In Vivo* Efficacy of Hypoxic Cytotoxin Tirapazamine and Hypoxic Cell Radiosensitizer Ku-2285 in Combination With Single and Fractionated Irradiation. Jpn J Cancer Res (1996) 87(1):98–104. 10.1111/j.1349-7006.1996.tb00206.x 8609056 PMC5920986

[36] Friery OP Hejmadi MV McKeown SR. Dna Damage Induced in T50/80 Tumour Cells Following Exposure to the Bioreductive Drug Tirapazamine in Combination With a Single Dose of Radiation (12gy). Biochem Soc Trans (1997) 25(1):135S. 10.1042/bst025135s 9057033

[37] Zhang M Stevens G. Effect of Radiation and Tirapazamine (Sr-4233) on Three Melanoma Cell Lines. Melanoma Res (1998) 8(6):510–5. 10.1097/00008390-199812000-00006 9918413

[38] Masunaga SI Tano K Sanada Y Sakurai Y Tanaka H Suzuki M. Effect of Tirapazamine, Metformin or Mild Hyperthermia on Recovery From Radiation-Induced Damage in Pimonidazole-Unlabeled Quiescent Tumor Cells. World J Oncol (2017) 8(5):137–46. 10.14740/wjon1058w PMC5687893 29147450

[39] Cliffe S Taylor ML Rutland M Baguley BC Hill RP Wilson WR. Combining Bioreductive Drugs (SR 4233 or SN 23862) With the Vasoactive Agents Flavone Acetic Acid or 5,6-Dimethylxanthenone Acetic Acid. Int J Radiat Oncol Biol Phys (1994) 29(2):373–7. 10.1016/0360-3016(94)90292-5 8195036

[40] Masunaga S Ono K Hori H Shibata T Suzuki M Kinashi Y. Effects of Bioreductive Agents, Tirapazamine and Mitomycin C, on Quiescent Cell Populations in Solid Tumors, Evaluated by Micronucleus Assay. Jpn J Cancer Res (1997) 88(9):907–14. 10.1111/j.1349-7006.1997.tb00468.x PMC5921522 9369940

[41] Weitman S Mangold G Marty J Dexter D Hilsenbeck S Rake J. Evidence of Enhanced *In Vivo* Activity Using Tirapazamine With Paclitaxel and Paraplatin Regimens Against the MV-522 Human Lung Cancer Xenograft. Cancer Chemother Pharmacol (1999) 43(5):402–8. 10.1007/s002800050914 10100596

[42] Jounaidi Y Waxman DJ. Combination of the Bioreductive Drug Tirapazamine With the Chemotherapeutic Prodrug Cyclophosphamide for P450/P450-Reductase-Based Cancer Gene Therapy. Cancer Res (2000) 60(14):3761–9. 10.1016/S0165-4608(00)00214-4 10919648

[43] Masunaga S Ono K Hori H Suzuki M Kinashi Y Takagaki M. Change in Oxygenation Status in Intratumour Total and Quiescent Cells Following Gamma-Ray Irradiation, Tirapazamine Administration, Cisplatin Injection and Bleomycin Treatment. Br J Radiol (2000) 73(873):978–86. 10.1259/bjr.73.873.11064652 11064652

[44] Doloff JC Khan N Ma J Demidenko E Swartz HM Jounaidi Y. Increased Tumor Oxygenation and Drug Uptake During Anti-Angiogenic Weekly Low Dose Cyclophosphamide Enhances the Anti-Tumor Effect of Weekly Tirapazamine. Curr Cancer Drug Targets (2009) 9(6):777–88. 10.2174/156800909789271503 PMC2912211 19754361

[45] Wilder RB Langmuir VK Mendonca HL Goris ML Knox SJ. Local Hyperthermia and SR 4233 Enhance the Antitumor Effects of Radioimmunotherapy in Nude Mice With Human Colonic Adenocarcinoma Xenografts. Cancer Res (1993) 53(13):3022–7. 10.1007/BF01517047 8319209

[46] Masunaga S Nagasawa H Uto Y Hori H Nagata K Suzuki M. The Usefulness of Mild Temperature Hyperthermia Combined With Continuous Tirapazamine Administration Under Reduced Dose-Rate Irradiation With Gamma-Rays. Int J Hyperthermia (2007) 23(1):29–35. 10.1080/02656730601135366 17575721

[47] Masunaga S Liu Y Sakurai Y Tanaka H Suzuki M Kondo N. Usefulness of Combined Treatment With Continuous Administration of Tirapazamine and Mild Temperature Hyperthermia in γ-Ray Irradiation in Terms of Local Tumour Response and Lung Metastatic Potential. Int J Hyperthermia (2012) 28(7):636–44. 10.3109/02656736.2012.714517 22946564

[48] Broekgaarden M Weijer R van Wijk AC Cox RC Egmond MR Hoebe R. Photodynamic Therapy With Liposomal Zinc Phthalocyanine and Tirapazamine Increases Tumor Cell Death *Via* DNA Damage. J BioMed Nanotechnol (2017) 13(2):204–20. 10.1166/jbn.2017.2327 29377650

[49] Lin WH Yeh SH Yeh KH Chen KW Cheng YW Su TH. Hypoxia-Activated Cytotoxic Agent Tirapazamine Enhances Hepatic Artery Ligation-Induced Killing of Liver Tumor in HBx Transgenic Mice. Proc Natl Acad Sci USA (2016) 113(42):11937–42. 10.1073/pnas.1613466113 PMC5081602 27702890

[50] Adam M Ottenjann S Künzel G Busch R Erhardt W Nieder C. Evaluation of the Toxicity of Tirapazamine Plus Cisplatin in a Mouse Tumor Model. Strahlenther Onkol (2006) 182(4):231–9. 10.1007/s00066-006-1506-z 16622625

[51] Adam M Bayer C Henke J Grosu A Molls M Nieder C. Tirapazamine Plus Cisplatin and Irradiation in a Mouse Model: Improved Tumor Control at the Cost of Increased Toxicity. J Cancer Res Clin Oncol (2008) 134(2):137–46. 10.1007/s00432-007-0260-7 PMC12161668 17622558

[52] Johnson CA Kilpatrick D von Roemeling R Langer C Graham MA Greenslade D. Phase I Trial of Tirapazamine in Combination With Cisplatin in a Single Dose Every 3 Weeks in Patients With Solid Tumors. J Clin Oncol (1997) 15(2):773–80. 10.1200/JCO.1997.15.2.773 9053504

[53] Hoff PM Saad ED Ravandi-Kashani F Czerny E Pazdur R. Phase I Trial of I.V. Administered Tirapazamine Plus Cyclophosphamide. Anticancer Drugs (2001) 12(6):499–503. 10.1097/00001813-200107000-00002 11459995

[54] Lara PN Jr Frankel P Mack PC Gumerlock PH Galvin I Martel CL. Tirapazamine Plus Carboplatin and Paclitaxel in Advanced Malignant Solid Tumors: A California Cancer Consortium Phase I and Molecular Correlative Study. Clin Cancer Res (2003) 9(12):4356–62. 10.1093/carcin/bgg164 14555506

[55] Aquino VM Weitman SD Winick NJ Blaney S Furman WL Kepner JL. Phase I Trial of Tirapazamine and Cyclophosphamide in Children With Refractory Solid Tumors: A Pediatric Oncology Group Study. J Clin Oncol (2004) 22(8):1413–9. 10.1200/JCO.2004.07.111 15084615

[56] Cohen EE Rosine D Haraf DJ Loh E Shen L Lusinchi A. Phase I Trial of Tirapazamine, Cisplatin, and Concurrent Accelerated Boost Reirradiation in Patients With Recurrent Head and Neck Cancer. Int J Radiat Oncol Biol Phys (2007) 67(3):678–84. 10.1016/j.ijrobp.2006.09.056 17293229

[57] Smith HO Jiang CS Weiss GR Hallum AV 3rd Liu PY Robinson WR 3rd. Tirapazamine Plus Cisplatin in Advanced or Recurrent Carcinoma of the Uterine Cervix: A Southwest Oncology Group Study. Int J Gynecol Cancer (2006) 16(1):298–305. 10.1111/j.1525-1438.2006.00339.x 16445649

[58] Reck M von Pawel J Nimmermann C Groth G Gatzemeier U. Phase II-trial of Tirapazamine in Combination With Cisplatin and Gemcitabine in Patients With Advanced Non-Small-Cell-Lung-Cancer (NSCLC). Pneumologie (2004) 58(12):845–9. 10.1055/s-2004-830056 15597251

[59] Covens A Blessing J Bender D Mannel R Morgan M. Gynecologic Oncology Group. A Phase II Evaluation of Tirapazamine Plus Cisplatin in the Treatment of Recurrent Platinum-Sensitive Ovarian or Primary Peritoneal Cancer: A Gynecologic Oncology Group Study. Gynecol Oncol (2006) 100(3):586–90. 10.1016/j.ygyno.2005.09.032 16249022

[60] Maluf FC Leiser AL Aghajanian C Sabbatini P Pezzulli S Chi DS. Phase II Study of Tirapazamine Plus Cisplatin in Patients With Advanced or Recurrent Cervical Cancer. Int J Gynecol Cancer (2006) 16(3):1165–71. 10.1111/j.1525-1438.2006.00454.x 16803501

[61] Ghatage P Sabagh H. Is There a Role for Tirapazamine in the Treatment of Cervical Cancer? Expert Opin Drug Metab Toxicol (2012) 8(12):1589–97. 10.1517/17425255.2012.730518 23033890

[62] Rischin D Narayan K Oza AM Mileshkin L Bernshaw D Choi J. Phase 1 Study of Tirapazamine in Combination With Radiation and Weekly Cisplatin in Patients With Locally Advanced Cervical Cancer. Int J Gynecol Cancer (2010) 20(5):827–33. 10.1111/IGC.0b013e3181dc827e 20606530

[63] Le QT Taira A Budenz S Jo Dorie M Goffinet DR Fee WE. Mature Results From a Randomized Phase II Trial of Cisplatin Plus 5-Fluorouracil and Radiotherapy With or Without Tirapazamine in Patients With Resectable Stage IV Head and Neck Squamous Cell Carcinomas. Cancer (2006) 106(9):1940–9. 10.1002/cncr.21785 16532436

[64] Williamson SK Crowley JJ Lara PN Jr McCoy J Lau DH Tucker RW. Phase III Trial of Paclitaxel Plus Carboplatin With or Without Tirapazamine in Advanced Non-Small-Cell Lung Cancer: Southwest Oncology Group Trial S0003. J Clin Oncol (2005) 23(36):9097–104. 10.1200/JCO.2005.01.3771 16361616

[65] Rischin D Peters LJ O’Sullivan B Giralt J Fisher R Yuen K. Tirapazamine, Cisplatin, and Radiation Versus Cisplatin and Radiation for Advanced Squamous Cell Carcinoma of the Head and Neck (Trog 02.02, Headstart): A Phase III Trial of the Trans-Tasman Radiation Oncology Group. J Clin Oncol (2010) 28(18):2989–95. 10.1200/JCO.2009.27.4449 20479425

[66] Patterson LH. Rationale for the Use of Aliphatic N-oxides of Cytotoxic Anthraquinones as Prodrug DNA Binding Agents: A New Class of Bioreductive Agent. Cancer Metastasis Rev (1993) 12(2):119–34. 10.1007/bf00689805 8375016

[67] McKeown SR Hejmadi MV McIntyre IA McAleer JJ Patterson LH. AQ4N: An Alkylaminoanthraquinone N-Oxide Showing Bioreductive Potential and Positive Interaction With Radiation *In Vivo* . Br J Cancer (1995) 72(1):76–81. 10.1038/bjc.1995.280 7599069 PMC2034137

[68] Hejmadi MV McKeown SR Friery OP McIntyre IA Patterson LH Hirst DG. DNA Damage Following Combination of Radiation With the Bioreductive Drug AQ4N: Possible Selective Toxicity to Oxic and Hypoxic Tumour Cells. Br J Cancer (1996) 73(4):499–505. 10.1038/bjc.1996.87 8595165 PMC2074454

[69] Patterson LH McKeown SR Ruparelia K Double JA Bibby MC Cole S. Enhancement of Chemotherapy and Radiotherapy of Murine Tumours by AQ4N, a Bioreductively Activated Anti-Tumour Agent. Br J Cancer (2000) 82(12):1984–90. 10.1054/bjoc.2000.1564 PMC2363261 10864207

[70] Gallagher R Hughes CM Murray MM Friery OP Patterson LH Hirst DG. The Chemopotentiation of Cisplatin by the Novel Bioreductive Drug AQ4N. Br J Cancer (2001) 85(4):625–9. 10.1054/bjoc.2001.1975 PMC2364091 11506506

[71] McCarthy HO Yakkundi A McErlane V Hughes CM Keilty G Murray M. Bioreductive GDEPT Using Cytochrome P450 3A4 in Combination With AQ4N. Cancer Gene Ther (2003) 10(1):40–8. 10.1038/sj.cgt.7700522 12489027

[72] Nishida CR Lee M de Montellano PR. Efficient Hypoxic Activation of the Anticancer Agent AQ4N by CYP2S1 and CYP2W1. Mol Pharmacol (2010) 78(3):497–502. 10.1124/mol.110.065045 20566689 PMC2939484

[73] Xiao Y Shinkyo R Guengerich FP. Cytochrome P450 2S1 Is Reduced by NADPH-cytochrome P450 Reductase. Drug Metab Dispos (2011) 39(6):944–6. 10.1124/dmd.111.039321 PMC3100906 21430234

[74] Bebenek IG Solaimani P Bui P Hankinson O. CYP2S1 Is Negatively Regulated by Corticosteroids in Human Cell Lines. Toxicol Lett (2012) 209(1):30–4. 10.1016/j.toxlet.2011.11.020 22155357

[75] Mehibel M Singh S Cowen RL Williams KJ Stratford IJ. Radiation Enhances the Therapeutic Effect of Banoxantrone in Hypoxic Tumour Cells With Elevated Levels of Nitric Oxide Synthase. Oncol Rep (2016) 35(4):1925–32. 10.3892/or.2016.4555 PMC4774668 26782976

[76] Lalani AS Alters SE Wong A Albertella MR Cleland JL Henner WD. Selective Tumor Targeting by the Hypoxia-Activated Prodrug AQ4N Blocks Tumor Growth and Metastasis in Preclinical Models of Pancreatic Cancer. Clin Cancer Res (2007) 13(7):2216–25. 10.1158/1078-0432.CCR-06-2427 17404106

[77] Williams KJ Albertella MR Fitzpatrick B Loadman PM Shnyder SD Chinje EC. *In Vivo* Activation of the Hypoxia-Targeted Cytotoxin AQ4N in Human Tumor Xenografts. Mol Cancer Ther (2009) 8(12):3266–75. 10.1158/1535-7163.MCT-09-0396 19996276

[78] Ming L Byrne NM Camac SN Mitchell CA Ward C Waugh DJ. Androgen Deprivation Results in Time-Dependent Hypoxia in LNCaP Prostate Tumours: Informed Scheduling of the Bioreductive Drug AQ4N Improves Treatment Response. Int J Cancer (2013) 132(6):1323–32. 10.1002/ijc.27796 22915157

[79] Manley E Jr Waxman DJ. Impact of Tumor Blood Flow Modulation on Tumor Sensitivity to the Bioreductive Drug Banoxantrone. J Pharmacol Exp Ther (2013) 344(2):368–77. 10.1124/jpet.112.200089 PMC3558827 23192656

[80] Gieling RG Fitzmaurice RJ Telfer BA Babur M Williams KJ. Dissemination *Via* the Lymphatic or Angiogenic Route Impacts the Pathology, Microenvironment and Hypoxia-Related Drug Response of Lung Metastases. Clin Exp Metastasis (2015) 32(6):567–77. 10.1007/s10585-015-9728-z 26112891

[81] Trédan O Garbens AB Lalani AS Tannock IF. The Hypoxia-Activated ProDrug AQ4N Penetrates Deeply in Tumor Tissues and Complements the Limited Distribution of Mitoxantrone. Cancer Res (2009) 69(3):940–7. 10.1158/0008-5472.CAN-08-0676 19176397

[82] Raghava S Kompella UB. AQ4, an Antitumor Anthracenedione, Inhibits Endothelial Cell Proliferation and Vascular Endothelial Growth Factor Secretion: Implications for the Therapy of Ocular Neovascular Disorders. Eur J Pharmacol (2007) 568(1-3):68–74. 10.1016/j.ejphar.2007.04.044 17543940 PMC6349398

[83] O’Rourke M Ward C Worthington J McKenna J Valentine A Robson T. Evaluation of the Antiangiogenic Potential of AQ4N. Clin Cancer Res (2008) 14(5):1502–9. 10.1158/1078-0432.CCR-07-1262 18316575

[84] Steward WP Middleton M Benghiat A Loadman PM Hayward C Waller S. The Use of Pharmacokinetic and Pharmacodynamic End Points to Determine the Dose of AQ4N, a Novel Hypoxic Cell Cytotoxin, Given With Fractionated Radiotherapy in a Phase I Study. Ann Oncol (2007) 18(6):1098–103. 10.1093/annonc/mdm120 17442658

[85] Albertella MR Loadman PM Jones PH Phillips RM Rampling R Burnet N. Hypoxia-Selective Targeting by the Bioreductive Prodrug AQ4N in Patients With Solid Tumors: Results of a Phase I Study. Clin Cancer Res (2008) 14(4):1096–104. 10.1158/1078-0432.CCR-07-4020 18281542

[86] Papadopoulos KP Goel S Beeram M Wong A Desai K Haigentz M. A Phase 1 Open-Label, Accelerated Dose-Escalation Study of the Hypoxia-Activated Prodrug AQ4N in Patients With Advanced Malignancies. Clin Cancer Res (2008) 14(21):7110–5. 10.1158/1078-0432.CCR-08-0483 18981010

[87] Shen S Wu Y Li K Wang Y Wu J Zeng Y. Versatile Hyaluronic Acid Modified AQ4N-Cu(II)-gossypol Infinite Coordination Polymer Nanoparticles: Multiple Tumor Targeting, Highly Efficient Synergistic Chemotherapy, and Real-Time Self-Monitoring. Biomaterials (2018) 154:197–212. 10.1016/j.biomaterials.2017.11.001 29128847

[88] Zhang D Wu M Cai Z Liao N Ke K Liu H. Chemotherapeutic Drug Based Metal-Organic Particles for Microvesicle-Mediated Deep Penetration and Programmable Ph/NIR/Hypoxia Activated Cancer Photochemotherapy. Adv Sci (Weinh) (2018) 5(2):1700648. 10.1002/advs.201700648 29619314 PMC5827097

[89] Luan X Guan YY Liu HJ Lu Q Zhao M Sun D. A Tumor Vascular-Targeted Interlocking Trimodal Nanosystem That Induces and Exploits Hypoxia. Adv Sci (Weinh) (2018) 5(8):1800034. 10.1002/advs.201800034 30128230 PMC6097144

[90] He Z Dai Y Li X Guo D Liu Y Huang X. Hybrid Nanomedicine Fabricated From Photosensitizer-Terminated Metal-Organic Framework Nanoparticles for Photodynamic Therapy and Hypoxia-Activated Cascade Chemotherapy. Small (2019) 15(4):e1804131. 10.1002/smll.201804131 30565431

[91] Li X Zhao Y Jiang W Li S Zhan M Liu H. Ultralong Circulating Choline Phosphate Liposomal Nanomedicines for Cascaded Chemo-Radiotherapy. Biomater Sci (2019) 7(4):1335–44. 10.1039/c9bm00051h 30816393

[92] Ji Y Lu F Hu W Zhao H Tang Y Li B. Tandem Activated Photodynamic and Chemotherapy: Using pH-Sensitive Nanosystems to Realize Different Tumour Distributions of Photosensitizer/Prodrug for Amplified Combination Therapy. Biomaterials (2019) 219:119393. 10.1016/j.biomaterials.2019.119393 31382206

[93] Feng L Cheng L Dong Z Tao D Barnhart TE Cai W. Theranostic Liposomes With Hypoxia-Activated Prodrug to Effectively Destruct Hypoxic Tumors Post-Photodynamic Therapy. ACS Nano (2017) 11(1):927–37. 10.1021/acsnano.6b07525 PMC5372701 28027442

[94] Zhang R Feng L Dong Z Wang L Liang C Chen J. Glucose & Oxygen Exhausting Liposomes for Combined Cancer Starvation and Hypoxia-Activated Therapy. Biomaterials (2018) 162:123–31. 10.1016/j.biomaterials.2018.02.004 29438880

[95] Singleton RS Guise CP Ferry DM Pullen SM Dorie MJ Brown JM. DNA Cross-Links in Human Tumor Cells Exposed to the Prodrug PR-104A: Relationships to Hypoxia, Bioreductive Metabolism, and Cytotoxicity. Cancer Res (2009) 69(9):3884–91. 10.1158/0008-5472.CAN-08-4023 19366798

[96] Guise CP Wang AT Theil A Bridewell DJ Wilson WR Patterson AV. Identification of Human Reductases That Activate the Dinitrobenzamide Mustard Prodrug PR-104A: A Role for NADPH:cytochrome P450 Oxidoreductase Under Hypoxia. Biochem Pharmacol (2007) 74(6):810–20. 10.1016/j.bcp.2007.06.014 17645874

[97] Guise CP Abbattista MR Singleton RS Holford SD Connolly J Dachs GU. The Bioreductive Prodrug PR-104A Is Activated Under Aerobic Conditions by Human Aldo-Keto Reductase 1C3. Cancer Res (2010) 70(4):1573–84. 10.1158/0008-5472.CAN-09-3237 20145130

[98] Gu Y Guise CP Patel K Abbattista MR Li J Sun X. Reductive Metabolism of the Dinitrobenzamide Mustard Anticancer Prodrug PR-104 in Mice. Cancer Chemother Pharmacol (2011) 67(3):543–55. 10.1007/s00280-010-1354-5 20473609

[99] Gu Y Patterson AV Atwell GJ Chernikova SB Brown JM Thompson LH. Roles of DNA Repair and Reductase Activity in the Cytotoxicity of the Hypoxia-Activated Dinitrobenzamide Mustard PR-104A. Mol Cancer Ther (2009) 8(6):1714–23. 10.1158/1535-7163.MCT-08-1209 19509245

[100] Hicks KO Myint H Patterson AV Pruijn FB Siim BG Patel K. Oxygen Dependence and Extravascular Transport of Hypoxia-Activated Prodrugs: Comparison of the Dinitrobenzamide Mustard PR-104A and Tirapazamine. Int J Radiat Oncol Biol Phys (2007) 69(2):560–71. 10.1016/j.ijrobp.2007.05.049 17869669

[101] Foehrenbacher A Patel K Abbattista MR Guise CP Secomb TW Wilson WR. The Role of Bystander Effects in the Antitumor Activity of the Hypoxia-Activated Prodrug Pr-104. Front Oncol (2013) 3:263. 10.3389/fonc.2013.00263 24109591 PMC3791487

[102] Cairns RA Bennewith KL Graves EE Giaccia AJ Chang DT Denko NC. Pharmacologically Increased Tumor Hypoxia Can be Measured by 18F-Fluoroazomycin Arabinoside Positron Emission Tomography and Enhances Tumor Response to Hypoxic Cytotoxin PR-104. Clin Cancer Res (2009) 15(23):7170–4. 10.1158/1078-0432.CCR-09-1676 PMC2810128 19920111

[103] Graves EE Vilalta M Cecic IK Erler JT Tran PT Felsher D. Hypoxia in Models of Lung Cancer: Implications for Targeted Therapeutics. Clin Cancer Res (2010) 16(19):4843–52. 10.1158/1078-0432.CCR-10-1206 PMC2948600 20858837

[104] Abbattista MR Jamieson SM Gu Y Nickel JE Pullen SM Patterson AV. Pre-Clinical Activity of PR-104 as Monotherapy and in Combination With Sorafenib in Hepatocellular Carcinoma. Cancer Biol Ther (2015) 16(4):610–22. 10.1080/15384047.2015.1017171 PMC4622463 25869917

[105] Jameson MB Rischin D Pegram M Gutheil J Patterson AV Denny WA. A Phase I Trial of PR-104, a Nitrogen Mustard Prodrug Activated by Both Hypoxia and Aldo-Keto Reductase 1C3, in Patients With Solid Tumors. Cancer Chemother Pharmacol (2010) 65(4):791–801. 10.1007/s00280-009-1188-1 20012293

[106] McKeage MJ Gu Y Wilson WR Hill A Amies K Melink TJ. A Phase I Trial of PR-104, a Pre-Prodrug of the Bioreductive Prodrug PR-104A, Given Weekly to Solid Tumour Patients. BMC Cancer (2011) 11:432. 10.1186/1471-2407-11-432 21982454 PMC3205073

[107] McKeage MJ Jameson MB Ramanathan RK Rajendran J Gu Y Wilson WR. Pr-104 a Bioreductive Pre-Prodrug Combined With Gemcitabine or Docetaxel in a Phase Ib Study of Patients With Advanced Solid Tumours. BMC Cancer (2012) 12:496. 10.1186/1471-2407-12-496 23098625 PMC3495895

[108] Abou-Alfa GK Chan SL Lin CC Chiorean EG Holcombe RF Mulcahy MF. Pr-104 Plus Sorafenib in Patients With Advanced Hepatocellular Carcinoma. Cancer Chemother Pharmacol (2011) 68(2):539–45. 10.1007/s00280-011-1671-3 21594722

[109] Benito J Shi Y Szymanska B Carol H Boehm I Lu H. Pronounced Hypoxia in Models of Murine and Human Leukemia: High Efficacy of Hypoxia-Activated Prodrug PR-104. PLoS One (2011) 6(8):e23108. 10.1371/journal.pone.0023108 21853076 PMC3154919

[110] Konopleva M Thall PF Yi CA Borthakur G Coveler A Bueso-Ramos C. Phase I/II Study of the Hypoxia-Activated Prodrug PR104 in Refractory/Relapsed Acute Myeloid Leukemia and Acute Lymphoblastic Leukemia. Haematologica (2015) 100(7):927–34. 10.3324/haematol.2014.118455 PMC4486227 25682597

[111] Moradi Manesh D El-Hoss J Evans K Richmond J Toscan CE Bracken LS. AKR1C3 Is a Biomarker of Sensitivity to PR-104 in Preclinical Models of T-Cell Acute Lymphoblastic Leukemia. Blood (2015) 126(10):1193–202. 10.1182/blood-2014-12-618900 PMC4559932 26116659

[112] Walton MI Sugget N Workman P. The Role of Human and Rodent DT-Diaphorase in the Reductive Metabolism of Hypoxic Cell Cytotoxins. Int J Radiat Oncol Biol Phys (1992) 22(4):643–7. 10.1016/0360-3016(92)90495-4 1544831

[113] Smitskamp-Wilms E Giaccone G Pinedo HM van der Laan BF Peters GJ. DT-Diaphorase Activity in Normal and Neoplastic Human Tissues; an Indicator for Sensitivity to Bioreductive Agents? Br J Cancer (1995) 72(4):917–21. 10.1038/bjc.1995.433 PMC2034035 7547240

[114] Fitzsimmons SA Workman P Grever M Paull K Camalier R Lewis AD. Reductase Enzyme Expression Across the National Cancer Institute Tumor Cell Line Panel: Correlation With Sensitivity to Mitomycin C and EO9. J Natl Cancer Inst (1996) 88(5):259–69. 10.1093/jnci/88.5.259 8614004

[115] Smitskamp-Wilms E Peters GJ Pinedo HM van Ark-Otte J Giaccone G. Chemosensitivity to the Indoloquinone EO9 Is Correlated With DT-diaphorase Activity and Its Gene Expression. Biochem Pharmacol (1994) 47(8):1325–32. 10.1016/0006-2952(94)90330-1 7514407

[116] Hendriks HR Pizao PE Berger DP Kooistra KL Bibby MC Boven E. EO9: A Novel Bioreductive Alkylating Indoloquinone With Preferential Solid Tumour Activity and Lack of Bone Marrow Toxicity in Preclinical Models. Eur J Cancer (1993) 29A(6):897–906. 10.1016/s0959-8049(05)80434-4 8484984

[117] Roed H Aabo K Vindeløv L Spang-Thomsen M Christensen IB Hansen HH. *In Vitro* and *In Vivo* Evaluation of the Indoloquinone EO-9 (NSC 382 459) Against Human Small Cell Carcinoma of the Lung. Eur J Cancer Clin Oncol (1989) 25(8):1197–201. 10.1016/0277-5379(89)90415-x 2548870

[118] Srivastava G Somasundaram RT Walfish PG Ralhan R. Anticancer Activity of Apaziquone in Oral Cancer Cells and Xenograft Model: Implications for Oral Cancer Therapy. PLoS One (2015) 10(7):e0133735. 10.1371/journal.pone.0133735 26208303 PMC4514673

[119] Begleiter A Leith MK Curphey TJ Doherty GP. Induction of DT-diaphorase in Cancer Chemoprevention and Chemotherapy. Oncol Res (1997) 9(6-7):371–82. 10.1016/0031-9422(91)83448-T 9406243

[120] Doherty GP Leith MK Wang X Curphey TJ Begleiter A. Induction of DT-diaphorase by 1,2-dithiole-3-thiones in Human Tumour and Normal Cells and Effect on Anti-Tumour Activity of Bioreductive Agents. Br J Cancer (1998) 77(8):1241–52. 10.1038/bjc.1998.209 PMC2150177 9579829

[121] Collard J Matthew AM Double JA Bibby MC. EO9: Relationship Between DT-Diaphorase Levels and Response *In Vitro* and *In Vivo* . Br J Cancer (1995) 71(6):1199–203. 10.1038/bjc.1995.233 PMC2033862 7779711

[122] Plumb JA Workman P. Unusually Marked Hypoxic Sensitization to Indoloquinone EO9 and Mitomycin C in a Human Colon-Tumour Cell Line That Lacks DT-diaphorase Activity. Int J Cancer (1994) 56(1):134–9. 10.1002/ijc.2910560124 8262670

[123] Bailey SM Wyatt MD Friedlos F Hartley JA Knox RJ Lewis AD. Involvement of DT-diaphorase (Ec 1.6.99.2) in the DNA Cross-Linking and Sequence Selectivity of the Bioreductive Anti-Tumour Agent EO9. Br J Cancer (1997) 76(12):1596–603. 10.1038/bjc.1997.603 PMC2228210 9413948

[124] Bando T Kasahara K Shibata K Numata Y Heki U Shirasaki H. Cytotoxicity of a Novel Indoloquinone Eo9 in Hypoxic Non-Small-Cell Lung-Cancer Cell-Lines. Int J Oncol (1995) 7(4):789–93. 10.3892/ijo.7.4.789 21552905

[125] Robertson N Haigh A Adams GE Stratford IJ. Factors Affecting Sensitivity to EO9 in Rodent and Human Tumour Cells *In Vitro*: DT-diaphorase Activity and Hypoxia. Eur J Cancer (1994) 30A(7):1013–9. 10.1016/0959-8049(94)90134-1 7946565

[126] Phillips RM Hulbert PB Bibby MC Sleigh NR Double JA. *In Vitro* Activity of the Novel Indoloquinone EO-9 and the Influence of Ph on Cytotoxicity. Br J Cancer (1992) 65(3):359–64. 10.1038/bjc.1992.73 PMC1977599 1558788

[127] McLeod HL Graham MA Aamdal S Setanoians A Groot Y Lund B. Phase I Pharmacokinetics and Limited Sampling Strategies for the Bioreductive Alkylating Drug EO9. Eortc Early Clinical Trials Group. Eur J Cancer (1996) 32A(9):1518–22. 10.1016/0959-8049(96)00120-7 8911111

[128] Aamdal S Lund B Koier I Houten M Wanders J Verweij J. Phase I Trial With Weekly EO9, a Novel Bioreductive Alkylating Indoloquinone, by the EORTC Early Clinical Study Group (Ecsg). Cancer Chemother Pharmacol (2000) 45:85–8. 10.1007/PL00006748 10647507

[129] Pavlidis N Hanauske AR Gamucci T Smyth J Lehnert M te Velde A. A Randomized Phase II Study With Two Schedules of the Novel Indoloquinone EO9 in Non-Small-Cell Lung Cancer: A Study of the EORTC Early Clinical Studies Group (Ecsg). Ann Oncol (1996) 7(5):529–31. 10.1093/oxfordjournals.annonc.a010645 8839911

[130] Dirix LY Tonnesen F Cassidy J Epelbaum R ten Bokkel Huinink WW Pavlidis N. EO9 Phase II Study in Advanced Breast, Gastric, Pancreatic and Colorectal Carcinoma by the EORTC Early Clinical Studies Group. Eur J Cancer (1996) 32A(11):2019–22. 10.1016/0959-8049(96)00226-2 8943690

[131] Schellens JH Dombernowsky P Cassidy J Epelbaum R Dirix L Cox EH. Population Pharmacokinetics and Dynamics in Phase II Studies of the Novel Bioreductive Alkylating Cytotoxic Indoloquinone EO9. Anticancer Drugs (2001) 12(7):583–90. 10.1097/00001813-200108000-00004 11487714

[132] Butler J Spanswick VJ Cummings J. The Autoxidation of the Reduced Forms of EO9. Free Radic Res (1996) 25(2):141–8. 10.3109/10715769609149919 8885332

[133] Phillips RM Hendriks HR Peters GJ. Eortc-Pharmacology and Molecular Mechanism Group. EO9 (Apaziquone): From the Clinic to the Laboratory and Back Again. Br J Pharmacol (2013) 168(1):11–8. 10.1111/j.1476-5381.2012.01996.x PMC3569998 22509926

[134] Phillips RM Hendriks HR Sweeney JB Reddy G Peters GJ. Efficacy, Pharmacokinetic and Pharmacodynamic Evaluation of Apaziquone in the Treatment of Non-Muscle Invasive Bladder Cancer. Expert Opin Drug Metab Toxicol (2017) 13(7):783–91. 10.1080/17425255.2017.1341490 28637373

[135] Caramés Masana F de Reijke TM. The Efficacy of Apaziquone in the Treatment of Bladder Cancer. Expert Opin Pharmacother (2017) 18(16):1781–8. 10.1080/14656566.2017.1392510 29034722

[136] Puri R Palit V Loadman PM Flannigan M Shah T Choudry GA. Phase I/II Pilot Study of Intravesical Apaziquone (EO9) for Superficial Bladder Cancer. J Urol (2006) 176(4 Pt 1):1344–8. 10.1016/j.juro.2006.06.047 16952628

[137] Karsh L Shore N Saltzstein D Bhat G Reddy G Allen LF. Integrated Results of Two Multicenter, Randomised, Placebo Controlled, Double Blind, Phase 3 Trials (SPI-611/612) of Single Dose Intravesical Apaziquone Immediately Following Resection in Patients With Nonmuscle Invasive Bladder Cancer. J Urol (2016) 95(Supplement):e290. 10.1016/j.juro.2016.02.847

[138] Witjes JA Karsh L Soloway M Bhat G Reddy G Allen Y. MP13-07 Improved Efficacy of Adjuvant, Single Dose Intravesical Apaziquone by Timing Post-Resection in Two Double Blind, Randomised, Placebo-Controlled Phase 3 Studies in Non-Muscle Invasive Bladder Cancer. J Urol (2016) 195(Supplement):e136. 10.1016/j.juro.2016.02.2488

[139] Karsh L Shore N Soloway M Bhat G Reddy G Leu SY. Double-Blind, Randomized, Placebo-Controlled Studies Evaluating Apaziquone (E09, Qapzola™) Intravesical Instillation Post Transurethral Resection of Bladder Tumors for the Treatment of Low-risk Non-Muscle Invasive Bladder Cancer. Bladder Cancer (2018) 4(3):293–301. 10.3233/BLC-180166 30112440 PMC6087454

[140] Phillips RM Loadman PM Reddy G. Inactivation of Apaziquone by Haematuria: Implications for the Design of Phase III Clinical Trials Against Non-Muscle Invasive Bladder Cancer. Cancer Chemother Pharmacol (2019) 83(6):1183–9. 10.1007/s00280-019-03812-7 PMC6499894 30868237

[141] Duan JX Jiao H Kaizerman J Stanton T Evans JW Lan L. Potent and Highly Selective Hypoxia-Activated Achiral Phosphoramidate Mustards as Anticancer Drugs. J Med Chem (2008) 51(8):2412–20. 10.1021/jm701028q 18257544

[142] Hunter FW Young RJ Shalev Z Vellanki RN Wang J Gu Y. Identification of P450 Oxidoreductase as a Major Determinant of Sensitivity to Hypoxia-Activated Prodrugs. Cancer Res (2015) 75(19):4211–23. 10.1158/0008-5472.CAN-15-1107 26297733

[143] Nytko KJ Grgic I Bender S Ott J Guckenberger M Riesterer O. The Hypoxia-Activated Prodrug Evofosfamide in Combination With Multiple Regimens of Radiotherapy. Oncotarget (2017) 8(14):23702–12. 10.18632/oncotarget.15784 PMC5410338 28423594

[144] Hu J Handisides DR Van Valckenborgh E De Raeve H Menu E Vande Broek I. Targeting the Multiple Myeloma Hypoxic Niche With Th-302, a Hypoxia-Activated Prodrug. Blood (2010) 116(9):1524–7. 10.1182/blood-2010-02-269126 20530289

[145] Liapis V Labrinidis A Zinonos I Hay S Ponomarev V Panagopoulos V. Hypoxia-Activated Pro-Drug Th-302 Exhibits Potent Tumor Suppressive Activity and Cooperates With Chemotherapy Against Osteosarcoma. Cancer Lett (2015) 357(1):160–9. 10.1016/j.canlet.2014.11.020 PMC4574867 25444931

[146] Voissiere A Jouberton E Maubert E Degoul F Peyrode C Chezal JM. Development and Characterization of a Human Three-Dimensional Chondrosarcoma Culture for *In Vitro* Drug Testing. PLoS One (2017) 12(7):e0181340. 10.1371/journal.pone.0181340 28704566 PMC5509331

[147] Zhang L Marrano P Wu B Kumar S Thorner P Baruchel S. Combined Antitumor Therapy With Metronomic Topotecan and Hypoxia-Activated Prodrug, Evofosfamide, in Neuroblastoma and Rhabdomyosarcoma Preclinical Models. Clin Cancer Res (2016) 22(11):2697–708. 10.1158/1078-0432.CCR-15-1853 26719428

[148] Stokes AM Hart CP Quarles CC. Hypoxia Imaging With PET Correlates With Antitumor Activity of the Hypoxia-Activated Prodrug Evofosfamide (Th-302) in Rodent Glioma Models. Tomography (2016) 2(3):229–37. 10.18383/j.tom.2016.00259 PMC5065246 27752544

[149] Liapis V Zinonos I Labrinidis A Hay S Ponomarev V Panagopoulos V. Anticancer Efficacy of the Hypoxia-Activated Prodrug Evofosfamide (TH-302) in Osteolytic Breast Cancer Murine Models. Cancer Med (2016) 5(3):534–45. 10.1002/cam4.599 PMC4799961 26749324

[150] Sun JD Liu Q Ahluwalia D Ferraro DJ Wang Y Jung D. Comparison of Hypoxia-Activated Prodrug Evofosfamide (TH-302) and Ifosfamide in Preclinical Non-Small Cell Lung Cancer Models. Cancer Biol Ther (2016) 17(4):371–80. 10.1080/15384047.2016.1139268 PMC5036787 26818215

[151] Huang Y Tian Y Zhao Y Xue C Zhan J Liu L. Efficacy of the Hypoxia-Activated Prodrug Evofosfamide (TH-302) in Nasopharyngeal Carcinoma *In Vitro* and *In Vivo* . Cancer Commun (Lond) (2018) 38(1):15. 10.1186/s40880-018-0285-0 29764490 PMC5993153

[152] Portwood S Lal D Hsu YC Vargas R Johnson MK Wetzler M. Activity of the Hypoxia-Activated Prodrug, TH-302, in Preclinical Human Acute Myeloid Leukemia Models. Clin Cancer Res (2013) 19(23):6506–19. 10.1158/1078-0432.CCR-13-0674 24088735

[153] Meng F Evans JW Bhupathi D Banica M Lan L Lorente G. Molecular and Cellular Pharmacology of the Hypoxia-Activated Prodrug Th-302. Mol Cancer Ther (2012) 11(3):740–51. 10.1158/1535-7163.MCT-11-0634 22147748

[154] Sun JD Liu Q Wang J Ahluwalia D Ferraro D Wang Y. Selective Tumor Hypoxia Targeting by Hypoxia-Activated Prodrug TH-302 Inhibits Tumor Growth in Preclinical Models of Cancer. Clin Cancer Res (2012) 18(3):758–70. 10.1158/1078-0432.CCR-11-1980 22184053

[155] Sun JD Liu Q Ahluwalia D Li W Meng F Wang Y. Efficacy and Safety of the Hypoxia-Activated Prodrug TH-302 in Combination With Gemcitabine and Nab-Paclitaxel in Human Tumor Xenograft Models of Pancreatic Cancer. Cancer Biol Ther (2015) 16(3):438–49. 10.1080/15384047.2014.100300 PMC4623012 25679067

[156] Ham SL Joshi R Luker GD Tavana H. Engineered Breast Cancer Cell Spheroids Reproduce Biologic Properties of Solid Tumors. Adv Healthc Mater (2016) 5(21):2788–98. 10.1002/adhm.201600644 PMC5142748 27603912

[157] Liapis V Zysk A DeNichilo M Zinonos I Hay S Panagopoulos V. Anticancer Efficacy of the Hypoxia-Activated Prodrug Evofosfamide Is Enhanced in Combination With Proapoptotic Receptor Agonists Against Osteosarcoma. Cancer Med (2017) 6(9):2164–76. 10.1002/cam4.1115 PMC5603834 28799237

[158] Saggar JK Tannock IF. Chemotherapy Rescues Hypoxic Tumor Cells and Induces Their Reoxygenation and Repopulation-An Effect That is Inhibited by the Hypoxia-Activated Prodrug Th-302. Clin Cancer Res (2015) 21(9):2107–14. 10.1158/1078-0432.CCR-14-2298 25677696

[159] Meng F Bhupathi D Sun JD Liu Q Ahluwalia D Wang Y. Enhancement of Hypoxia-Activated Prodrug TH-302 Anti-Tumor Activity by Chk1 Inhibition. BMC Cancer (2015) 15:422. 10.1186/s12885-015-1387-6 25994202 PMC4453293

[160] Sun JD Ahluwalia D Liu Q Li W Wang Y Meng F. Combination Treatment With Hypoxia-Activated Prodrug Evofosfamide (TH-302) and mTOR Inhibitors Results in Enhanced Antitumor Efficacy in Preclinical Renal Cell Carcinoma Models. Am J Cancer Res (2015) 5(7):2139–55. PMC4548326 26328245

[161] Wojtkowiak JW Cornnell HC Matsumoto S Saito K Takakusagi Y Dutta P. Pyruvate Sensitizes Pancreatic Tumors to Hypoxia-Activated Prodrug Th-302. Cancer Metab (2015) 3(1):2. 10.1186/s40170-014-0026-z 25635223 PMC4310189

[162] Takakusagi Y Kishimoto S Naz S Matsumoto S Saito K Hart CP. Radiotherapy Synergizes With the Hypoxia-Activated Prodrug Evofosfamide: *In Vitro* and *In Vivo* Studies. Antioxid Redox Signal (2018) 28(2):131–40. 10.1089/ars.2017.7106 PMC5725636 28741367

[163] Hajj C Russell J Hart CP Goodman KA Lowery MA Haimovitz-Friedman A. A Combination of Radiation and the Hypoxia-Activated Prodrug Evofosfamide (Th-302) is Efficacious Against a Human Orthotopic Pancreatic Tumor Model. Transl Oncol (2017) 10(5):760–5. 10.1016/j.tranon.2017.06.010 PMC5538966 28778024

[164] Spiegelberg L van Hoof SJ Biemans R Lieuwes NG Marcus D Niemans R. Evofosfamide Sensitizes Esophageal Carcinomas to Radiation Without Increasing Normal Tissue Toxicity. Radiother Oncol (2019) 141:247–55. 10.1016/j.radonc.2019.06.034 PMC6913516 31431383

[165] Duran R Mirpour S Pekurovsky V Ganapathy-Kanniappan S Brayton CF Cornish TC. Preclinical Benefit of Hypoxia-Activated Intra-Arterial Therapy With Evofosfamide in Liver Cancer. Clin Cancer Res (2017) 23(2):536–48. 10.1158/1078-0432.CCR-16-0725 PMC5241187 27440271

[166] Yoon C Chang KK Lee JH Tap WD Hart CP Simon MC. Multimodal Targeting of Tumor Vasculature and Cancer Stem-Like Cells in Sarcomas With VEGF-A Inhibition, HIF-1α Inhibition, and Hypoxia-Activated Chemotherapy. Oncotarget (2016) 7(28):42844–58. 10.18632/oncotarget PMC5189991 27374091

[167] Kumar S Sun JD Zhang L Mokhtari RB Wu B Meng F. Hypoxia-Targeting Drug Evofosfamide (Th-302) Enhances Sunitinib Activity in Neuroblastoma Xenograft Models. Transl Oncol (2018) 11(4):911–9. 10.1016/j.tranon.2018.05.004 PMC6041570 29803017

[168] Riedel RF Meadows KL Lee PH Morse MA Uronis HE Blobe GC. Phase I Study of Pazopanib Plus TH-302 in Advanced Solid Tumors. Cancer Chemother Pharmacol (2017) 79(3):611–9. 10.1007/s00280-017-3256-2 28238078

[169] Benito J Ramirez MS Millward NZ Velez J Harutyunyan KG Lu H. Hypoxia-Activated Prodrug Th-302 Targets Hypoxic Bone Marrow Niches in Preclinical Leukemia Models. Clin Cancer Res (2016) 22(7):1687–98. 10.1158/1078-0432.CCR-14-3378 PMC4818660 26603259

[170] Lindsay D Garvey CM Mumenthaler SM Foo J. Leveraging Hypoxia-Activated Prodrugs to Prevent Drug Resistance in Solid Tumors. PLoS Comput Biol (2016) 12(8):e1005077. 10.1371/journal.pcbi.1005077 27560187 PMC4999195

[171] Jamieson SM Tsai P Kondratyev MK Budhani P Liu A Senzer NN. Evofosfamide for the Treatment of Human Papillomavirus-Negative Head and Neck Squamous Cell Carcinoma. JCI Insight (2018) 3(16):e122204. 10.1172/jci.insight.122204 PMC6141174 30135316

[172] Jayaprakash P Ai M Liu A Budhani P Bartkowiak T Sheng J. Targeted Hypoxia Reduction Restores T Cell Infiltration and Sensitizes Prostate Cancer to Immunotherapy. J Clin Invest (2018) 128(11):5137–49. 10.1172/JCI96268 PMC6205399 30188869

[173] Kishimoto S Brender JR Chandramouli GVR Saida Y Yamamoto K Mitchell JB. Hypoxia-Activated Prodrug Evofosfamide Treatment in Pancreatic Ductal Adenocarcinoma Xenografts Alters the Tumor Redox Status to Potentiate Radiotherapy. Antioxid Redox Signal (2020). 10.1089/ars.2020.8131 PMC8568781 32787454

[174] Weiss GJ Infante JR Chiorean EG Borad MJ Bendell JC Molina JR. Phase 1 Study of the Safety, Tolerability, and Pharmacokinetics of TH-302, a Hypoxia-Activated Prodrug, in Patients With Advanced Solid Malignancies. Clin Cancer Res (2011) 17(9):2997–3004. 10.1158/1078-0432.CCR-10-3425 21415214

[175] Chawla SP Cranmer LD Van Tine BA Reed DR Okuno SH Butrynski JE. Phase II Study of the Safety and Antitumor Activity of the Hypoxia-Activated Prodrug TH-302 in Combination With Doxorubicin in Patients With Advanced Soft Tissue Sarcoma. J Clin Oncol (2014) 32(29):3299–306. 10.1200/JCO.2013.54.3660 PMC4588714 25185097

[176] Borad MJ Reddy SG Bahary N Uronis HE Sigal D Cohn AL. Randomized Phase II Trial of Gemcitabine Plus TH-302 *Versus* Gemcitabine in Patients With Advanced Pancreatic Cancer. J Clin Oncol (2015) 33(13):1475–81. 10.1200/JCO.2014.55.7504 PMC4881365 25512461

[177] Brenner A Zuniga R Sun JD Floyd J Hart CP Kroll S. Hypoxia-Activated Evofosfamide for Treatment of Recurrent Bevacizumab-Refractory Glioblastoma: A Phase I Surgical Study. Neuro Oncol (2018) 20(9):1231–9. 10.1093/neuonc/noy015 PMC6071657 29415215

[178] Laubach JP Liu CJ Raje NS Yee AJ Armand P Schlossman RL. A Phase I/Ii Study of Evofosfamide, A Hypoxia-Activated Prodrug With or Without Bortezomib in Subjects With Relapsed/Refractory Multiple Myeloma. Clin Cancer Res (2019) 25(2):478–86. 10.1158/1078-0432 PMC6335171 30279233

[179] Badar T Handisides DR Benito JM Richie MA Borthakur G Jabbour E. Phase I Study of Evofosfamide, an Investigational Hypoxia-Activated Prodrug, in Patients With Advanced Leukemia. Am J Hematol (2016) 91(8):800–5. 10.1002/ajh.24415 PMC4946992 27169385

[180] Van Cutsem E Lenz HJ Furuse J Tabernero J Heinemann V Ioka T. Maestro: A Randomized, Double-Blind Phase III Study of Evofosfamide (Evo) in Combination With Gemcitabine (Gem) in Previously Untreated Patients (Pts) With Metastatic or Locally Advanced Unresectable Pancreatic Ductal Adenocarcinoma (PDAC). J Clin Oncol (2016) 34(15_suppl):4007. 10.1200/JCO.2016.34.15_suppl.4007

[181] Tap WD Papai Z Van Tine BA Attia S Ganjoo KN Jones RL. Doxorubicin Plus Evofosfamide *Versus* Doxorubicin Alone in Locally Advanced, Unresectable or Metastatic Soft-Tissue Sarcoma (TH CR-406/SARC021): An International, Multicentre, Open-Label, Randomised Phase 3 Trial. Lancet Oncol (2017) 18(8):1089–103. 10.1016/S1470-2045(17)30381-9 PMC7771354 28651927

[182] Domenyuk V Liu X Magee D Gatalica Z Stark A Kennedy P. Poly-Ligand Profiling Differentiates Pancreatic Cancer Patients According to Treatment Benefit From Gemcitabine+Placebo *Versus* Gemcitabine+Evofosfamide and Identifies Candidate Targets. Ann Oncol (2018) 29(suppl_5):mdy151.131. 10.1093/annonc/mdy151.131

[183] Li Y Zhao L Li XF. The Hypoxia-Activated Prodrug TH-302: Exploiting Hypoxia in Cancer Therapy. Front Pharmacol (2021) 12:636892. 10.3389/fphar.2021.636892 33953675 PMC8091515

[184] Anderson RF Li D Hunter FW. Antagonism in Effectiveness of Evofosfamide and Doxorubicin Through Intermolecular Electron Transfer. Free Radic Biol Med (2017) 113:564–70. 10.1016/j.freeradbiomed.2017.10.385 29111232

[185] Higgins JP Sarapa N Kim J Poma E. Unexpected Pharmacokinetics of Evofosfamide Observed in Phase III MAESTRO Study. J Clin Oncol (2018) 36(15_suppl):2568. 10.1200/JCO.2018.36.15_suppl.2568

[186] Hunter FW Wang J Patel R Hsu HL Hickey AJ Hay MP. Homologous Recombination Repair-Dependent Cytotoxicity of the Benzotriazine Di-N-Oxide CEN-209: Comparison With Other Hypoxia-Activated Prodrugs. Biochem Pharmacol (2012) 83(5):574–85. 10.1016/j.bcp.2011.12.005 22182429

[187] Hicks KO Siim BG Jaiswal JK Pruijn FB Fraser AM Patel R. Pharmacokinetic/Pharmacodynamic Modeling Identifies SN30000 and SN29751 as Tirapazamine Analogues With Improved Tissue Penetration and Hypoxic Cell Killing in Tumors. Clin Cancer Res (2010) 16(20):4946–57. 10.1158/1078-0432.CCR-10-1439 PMC3390971 20732963

[188] Mao X McManaway S Jaiswal JK Patel PB Wilson WR Hicks KO. An Agent-Based Model for Drug-Radiation Interactions in the Tumour Microenvironment: Hypoxia-Activated Prodrug SN30000 in Multicellular Tumour Spheroids. PLoS Comput Biol (2018) 14(10):e1006469. 10.1371/journal.pcbi.1006469 30356233 PMC6218095

[189] Mao X McManaway S Jaiswal JK Hong CR Wilson WR Hicks KO. Schedule-Dependent Potentiation of Chemotherapy Drugs by the Hypoxia-Activated Prodrug Sn30000. Cancer Biol Ther (2019) 20(9):1258–69. 10.1080/15384047.2019.1617570 PMC6741573 31131698

[190] Chitneni SK Bida GT Yuan H Palmer GM Hay MP Melcher T. 18f-Ef5 PET Imaging as an Early Response Biomarker for the Hypoxia-Activated Prodrug SN30000 Combined With Radiation Treatment in a Non-Small Cell Lung Cancer Xenograft Model. J Nucl Med (2013) 54(8):1339–46. 10.2967/jnumed.112.116293 PMC3770943 23740105

[191] Wang J Foehrenbacher A Su J Patel R Hay MP Hicks KO. The 2-Nitroimidazole EF5 Is a Biomarker for Oxidoreductases That Activate the Bioreductive Prodrug CEN-209 Under Hypoxia. Clin Cancer Res (2012) 18(6):1684–95. 10.1158/1078-0432.CCR-11-2296 22167409

[192] Grkovski M Fanchon L Pillarsetty NVK Russell J Humm JL. 18F-Fluoromisonidazole Predicts Evofosfamide Uptake in Pancreatic Tumor Model. EJNMMI Res (2018) 8(1):53. 10.1186/s13550-018-0409-1 29916085 PMC6005997

[193] Cui YL Wang X Li XF. (18)F-Fluoromisonidazole PET Reveals Spatial and Temporal Heterogeneity of Hypoxia in Mouse Models of Human Non-Small-Cell Lung Cancer. Future Oncol (2015) 11(20):2841–9. 10.2217/fon.15.205 26361064

[194] Ljungkvist AS Bussink J Kaanders JH Rijken PF Begg AC Raleigh JA. Hypoxic Cell Turnover in Different Solid Tumor Lines. Int J Radiat Oncol Biol Phys (2005) 62(4):1157–68. 10.1016/j.ijrobp.2005.03.049 15913908

[195] Li XF Sun X Ma Y Suehiro M Zhang M Russell J. Detection of Hypoxia in Microscopic Tumors Using 131I-Labeled Iodo-Azomycin Galactopyranoside (131I-IAZGP) Digital Autoradiography. Eur J Nucl Med Mol Imaging (2010) 37(2):339–48. 10.1007/s00259-009-1310-y PMC2891169 19921184

[196] Li XF Ma Y Sun X Humm JL Ling CC O’Donoghue JA. High 18F-FDG Uptake in Microscopic Peritoneal Tumors Requires Physiologic Hypoxia. J Nucl Med (2010) 51(4):632–8. 10.2967/jnumed.109.071233 PMC2917184 20351353

[197] Huang T Civelek AC Zheng H Ng CK Duan X Li J. (18)F-Misonidazole PET Imaging of Hypoxia in Micrometastases and Macroscopic Xenografts of Human Non-Small Cell Lung Cancer: A Correlation With Autoradiography and Histological Findings. Am J Nucl Med Mol Imaging (2013) 3(2):142–53. PMC3601474 23526377

